# Whole-genome sequencing identifies *ADGRG6* enhancer mutations and *FRS2* duplications as angiogenesis-related drivers in bladder cancer

**DOI:** 10.1038/s41467-019-08576-5

**Published:** 2019-02-12

**Authors:** Song Wu, Tong Ou, Nianzeng Xing, Jiang Lu, Shengqing Wan, Changxi Wang, Xi Zhang, Feiya Yang, Yi Huang, Zhiming Cai

**Affiliations:** 1 Urology Institute of Shenzhen University, The Third Affiliated Hospital of Shenzhen University, Shenzhen University, Shenzhen, 518000 China; 2 Shenzhen Following Precision Medical Research Institute, Luohu Hospital Group, Shenzhen, 518000 China; 3 Department of Urology, Minimally Invasive Surgery Center, The First Affiliated Hospital of Guangzhou Medical University, Guangzhou, 510000 China; 4 Department of Urology, Beijing Chaoyang Hospital, Capital Medical University, Beijing, 100000 China; 5 Department of Urology, National Cancer Center/National Clinical Research Center for Cancer/Cancer Hospital, Chinese Academy of Medical Sciences and Peking Union Medical College, Beijing, 100000 China

## Abstract

Bladder cancer is one of the most common and highly vascularized cancers. To better understand its genomic structure and underlying etiology, we conduct whole-genome and targeted sequencing in urothelial bladder carcinomas (UBCs, the most common type of bladder cancer). Recurrent mutations in noncoding regions affecting gene regulatory elements and structural variations (SVs) leading to gene disruptions are prevalent. Notably, we find recurrent *ADGRG6* enhancer mutations and *FRS2* duplications which are associated with higher protein expression in the tumor and poor prognosis. Functional assays demonstrate that depletion of *ADGRG6* or *FRS2* expression in UBC cells compromise their abilities to recruit endothelial cells and induce tube formation. Moreover, pathway assessment reveals recurrent alterations in multiple angiogenesis-related genes. These results illustrate a multidimensional genomic landscape that highlights noncoding mutations and SVs in UBC tumorigenesis, and suggest ADGRG6 and FRS2 as novel pathological angiogenesis regulators that would facilitate vascular-targeted therapies for UBC.

## Abstract (web summary)

Bladder cancer is one of the most common and highly vascularized cancers. Here the authors perform a whole-genome analysis in urothelial bladder carcinomas and identify recurrent genetic alterations in a set of angiogenesis genes, facilitating the understanding of molecular mechanisms underlying pathological angiogenesis in this type of cancer.

## Introduction

Bladder cancer is a common genitourinary malignancy with an estimated 429,000 new cases and 165,000 deaths per year worldwide^1^, and no molecularly targeted anticancer agents have been approved for treatment of the complex disease. The majorities of bladder cancers (>90%) are urothelial bladder carcinomas (UBCs), which have been further classified into two clearly distinct groups, superficial nonmuscle-invasive bladder cancer (NMIBC) and MIBC, showing different clinical behavior^2,3^. UBC is a molecularly heterogeneous disease whose genome harbors various forms of somatic genetic alterations spanning from nucleotide-level mutations to large chromosomal changes. Recently, we and others reported genomic sequencing analyses of UBCs^4–6^, which mainly nominated cancer-associated genes driven by point mutations in protein-coding exons and copy-number changes. Whole-genome sequencing analyses on several other cancer types and recent pan-cancer analyses suggest that structural variations (SVs) and somatic mutations of noncoding regulatory regions could have crucial roles in carcinogenesis^7–10^. However, systematic analyses of noncoding mutations and SVs have not yet been performed for UBC.

Tumor angiogenesis, a pathophysiological process of new blood vessel formation in the primary tumor site or distant organs, is a classical hallmark of cancer and promotes tumor growth and progression by supplying sufficient nourishment to cancer cells and helping escaping tumor cells metastasize to distant sites^11,12^. Therefore, targeting tumor angiogenesis is an alternative approach for cancer therapy in combination with the direct attack of tumor cells. UBC is a highly vascularized cancer^13^, whereas its molecular basis and the involved signaling pathway remain largely uncharacterized. Detailed mechanistic insight into the relationship between pathological angiogenesis and genetic alternations are urgently required to appropriately utilize existing antiangiogenic drugs and provide novel targets for antiangiogenesis therapy in UBC.

In this study, using whole-genome sequencing in 65 UBCs and targeted sequencing in an additional 196 UBCs, we uncover the whole-genome mutational landscape of UBC and show that noncoding mutations and SVs have biological relevance and affect gene expression and signal transductions in regulation of tumor angiogenesis.

## Results

### Whole-genome sequencing of UBC samples

**Fig. 1:**
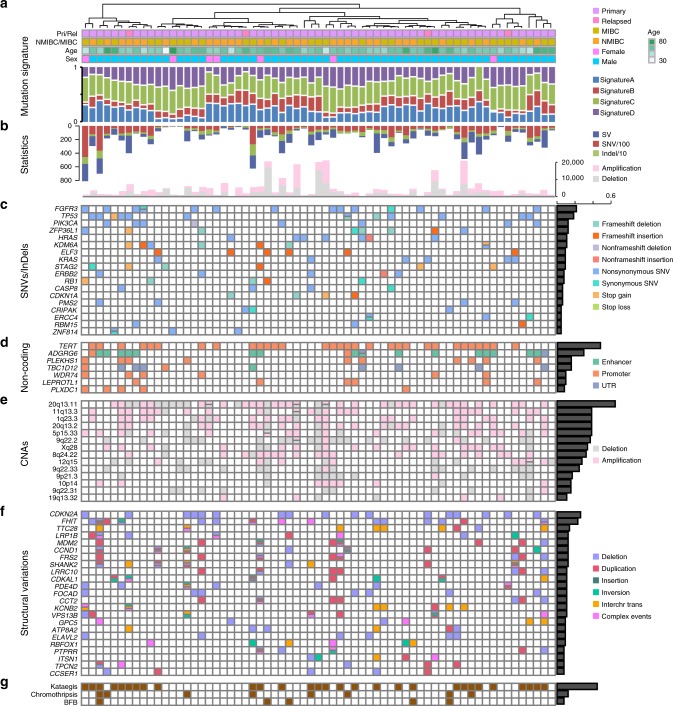
Multidimensional genomic mutational landscape in UBC. **a** Genome-wide mutational signatures and clinical features of 65 UBC cases. Four mutational signatures are identified and tumors are clustered based on the mutational signatures. **b** The total number of SVs/SNVs/indels (upper) and CNVs (bottom) in each case. The order of case in this part, as well as the following parts, is consistent with that in part a. **c** Significantly mutated genes altered by SNVs/Indels in coding regions. Mutation types are labeled with different colors which are annotated on the right legend, and the percentage of UBC tumors with the indicated gene mutation is noted on the right. **d** The seven most frequent genes with noncoding regulatory element mutations, including enhancer, promoter, and UTR mutations. **e** Recurrent focal regions of amplification (pink) and deletion (gray). **f** Significantly altered genes disrupted by SVs which are annotated on the left legend. **g** Genomes with catastrophes which are caused by chromothripsis, kataegis or BFB

We performed deep whole-genome sequencing of tumor and matched peripheral blood samples from 65 individuals with UBC, including 32 NMIBCs and 33 MIBCs. Clinical and pathological features are summarized (Supplementary Table 1 and Fig. 1a). After removal of polymerase chain reaction (PCR) duplicates, the average genome coverage was 37.4×, with 98.0% of the reference human genome covered by ≥4× (Supplementary Fig. 1). Single-nucleotide variations (SNVs), SVs, and insertions or deletions (indels) were called by several rigorous bioinformatic analysis steps (Online methods), and validations were carried out using custom liquid capture for candidate genetic alterations. In the combined discovery and validation cohorts, we identified an average of 8398.8 point mutations, 382.7 indels, and 82.9 SVs per sample (Supplementary Data 1 and Fig. 1b). In addition, the numbers of SNVs, SVs, and indels are uncorrelated with patient sex, age, and clinical phenotype (Supplementary Table 2).

### The mutational signatures of UBC

In examining the mutation spectrum, we applied nonnegative matrix factorization and identified four mutational signatures (Signatures A–D) in the UBC cohort (Supplementary Fig. 2). For validation of these signatures, we compared them to the signatures identified in Catalog of Somatic Mutations in Cancer^14^ (COSMIC) (Supplementary Table 3). Most signatures in our study showed high similarity to the COSMIC signatures, except for signature D, which was enriched C > A and T > A substitutions and could be a novel mutational signature. Associations of these four signatures with genetic alterations and clinical background were performed by multiple linear regression analysis (Supplementary Table 4).

### Recurrently mutated protein-coding genes

We examined the numbers of somatic substitutions and indels in protein-coding exons, and identified twenty significantly mutated genes, including previously known bladder cancer-associated oncogenes or tumor suppressors (e.g., *FGFR3*, *TP53*, and *PIK3CA*), and new significantly mutated genes (e.g., *CASP8*, *PMS2*, and *ZNF814*) (Fig. 1c). The frequencies of *ZFP36L1* and *ELF3* mutations were significantly higher in this UBC cohort than in previous UBC study or many other TCGA cancer type^4,6,7^. *ZFP36L1*, which encodes a RNA-binding protein regulating gene-expression post-transcriptionally by promoting AU-rich element-mediated mRNA decay and exerts DNA damage response and cell cycle regulation functions^15,16^, was mutated in 12.3% of UBC tumors. *ELF3*, encoding a transcriptional activator which binds to a purine-rich GGAA/T core motif in the target gene promoter and may play a significant role in epithelial cell fate determination^17^, was mutated 9.2% of UBC tumors. Both of them had a striking prevalence of deleterious missense mutations and frameshift insertions, which are highly consistent with tumor suppressor mutational inactivation patterns (Supplementary Fig. 3). We assessed the expression of *ZFP36L1* and *ELF3* in six UBC cell lines and an immortalized normal bladder urothelial cell line, and found that *ZFP36L1* and *ELF3* were lowly expressed in all UBC cell lines relative to normal control cell line (Supplementary Fig. 3).

### Recurrent noncoding mutations and their affected genes

**Fig. 2:**
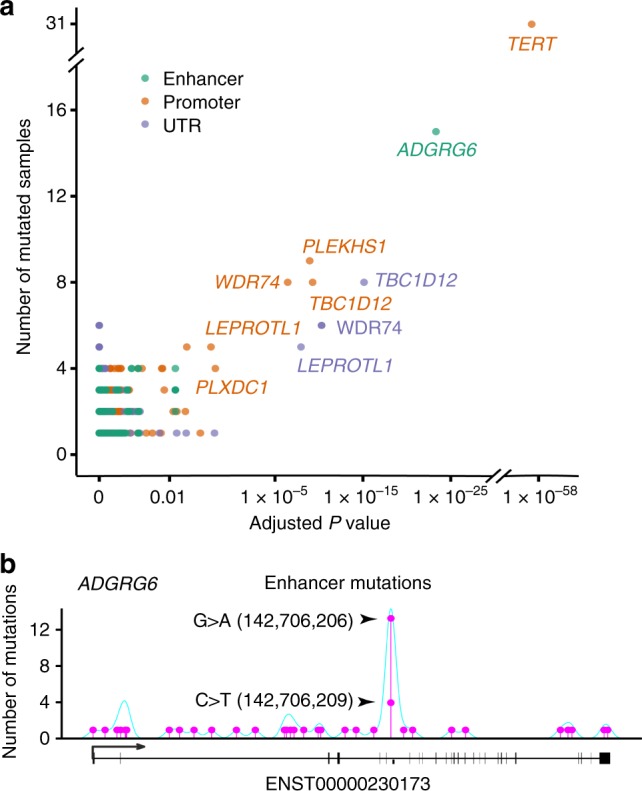
Characterization of recurrent noncoding regulatory mutations and their affected genes in UBC. **a** Significance of somatic mutations in noncoding regulatory regions. The number of samples (*y*-axis) which have point mutations and SV breakpoints in regulatory regions is plotted against the Benjamini–Hochberg adjusted *P* value (*x*-axis, binomial test) of each regulatory region comparing to its background region. Colors indicate the type of regulatory region. **b** Somatic mutations in the region of *ADGRG6*, including two highly recurrent sites (chr. 6: 142,706,206 G > A and chr. 6: 142,706,209 C > T; indicated by arrows) located in the enhancer region. Mutation density across the region is shown as a turquoise curve

The protein-coding component accounts for less than 2% of the total genomic sequence, but roughly 80% of human genome has been estimated to involve in some sort of biochemical networks^18^. There is very little information on how noncoding genetic alteration affects bladder cancer development, except for the recent discovery of mutations in the *TERT* promoter^19^. To investigate noncoding somatic driver mutations, we searched for noncoding genomic regions with more mutations than expected from chance. There were a number of regulatory regions with significant enrichment of noncoding mutations (Supplementary Table 5 and Fig. 2a). Among these regions, the *TERT* promoter exhibited the highest mutation frequency and showed the lowest *P* value, as reported in our previous study^19^. In addition, five promoters, including these of *PLEKHS1*, *TBC1D12*, *WDR74*, *LEPROTL1*, and *PLXDC1*, three UTRs, including those of *TBC1D12*, *WDR74*, and *LEPROTL1*, and *ADGRG6* enhancer, were identified as recurrently mutated noncoding elements (Fig. 2a). The five most frequent genes with noncoding regulatory element mutations were *TERT*, *ADGRG6*, *PLEKHS1*, *WDR74*, and *LEPROTL1*, and these genes affected 63% of UBC tumors (Fig. 1d).

**Fig. 3:**
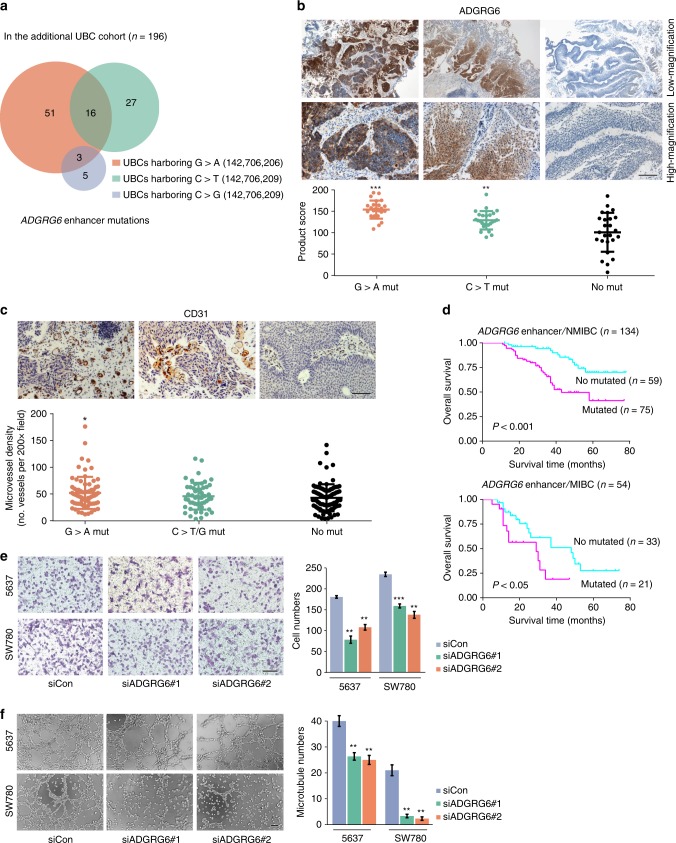
*ADGRG6* enhancer mutations are associated with poor survival and tumor angiogenesis in UBC. **a** Sanger sequencing detected *ADGRG6* enhancer mutations in the additional 196 UBCs. **b** Immunohistochemical staining of UBC tumors displaying consequences of *ADGRG6* enhancer mutations*. ADGRG6* enhancer mutations lead to ADGRG6 protein overexpression in tumor tissues. The three different groups of UBC tumors: G > A Mut (*n* = 25), *ADGRG6* enhancer only with 142,706,209 G > A mutation; C > T Mut (*n* = 25), *ADGRG6* enhancer only with 142,706,206C > T mutation; No Mut (*n* = 25), *ADGRG6* enhancer with no mutation. **c** CD31 immunostained microvessel in the additional 196 UBCs. The three different groups of UBC tumors: G > A Mut (*n* = 70), *ADGRG6* enhancer with 142,706,209G > A mutation; C > T/G Mut (*n* = 51), *ADGRG6* enhancer with 142,706,206 C > T/G mutation; No Mut (*n* = 94), *ADGRG6* enhancer with no mutation. The three horizontal lines represent mean ± SD for the subjects. *P < 0.05, **P < 0.01, ***P < 0.001 vs. No Mut group, two-sided unpaired *t* test with Welch’s correction (**b**) or Mann–Whitney test (**c**). **d** Kaplan–Meier survival curves show that the patients with the *ADGRG6* enhancer mutations were significantly associated with a shorter overall survival than those with no mutation in both nonmuscle-invasive bladder cancer (NMIBC) and muscle-invasive bladder cancer (MIBC) subcohorts. Statistical significance was determined by log-rank test. **e**, **f** Knockdown of ADGRG6 in 5637 and SW780 cells compromised their abilities to recruit human umbilical vein endothelial cells (**e**) and induce tube formation (**f**). Scale bars, 100 µm. Error bars represent the SEM. The data shown represent averages from three independent experiments and were statistically analyzed by two-sided *t* test. *P < 0.05, **P < 0.01, ***P < 0.001 vs. siCon

*ADGRG6* encodes a novel adhesion G protein-coupled receptor that is highly enriched in endothelial cells and plays an important role in angiogenesis^20^. The enhancer of *ADGRG6* exhibited recurrent mutations at two genomic positions, which were mutated in 13 (chr. 6: 142,706,206; G > A transition) and 4 (chr. 6: 142,706,209; C > T transition) samples (Fig. 2b). In an additional independent UBC cohort (*n* = 196, Supplementary Table 6), extracting paraffin-embedded tumor tissue DNA and Sanger sequencing the enhancer of *ADGRG6* detected the similar mutational spectrum and higher mutational incidence, which might be due to the increased purity of tumor cells in tissue sections and the enhanced accuracy of Sanger sequencing (Fig. 3a). *ADGRG6* enhancer mutation was positively correlated with older patients and nonmuscle-invasive tumors in the additional UBC cohort (*P* < 0.05; Supplementary Table 7). Moreover, we observed that UBC tumors with the *ADGRG6* enhancer mutations indeed showed significantly higher expression level of ADGRG6 than that in other samples without mutation as determined by immunohistochemistry analysis (Fig. 3b). Elevated microvessel density, as determined immunohistochemically using anti-CD31 monoclonal antibodies, was also significantly associated with the *ADGRG6* enhancer G > A mutation (chr. 6: 142,706,206), whereas there were no differences in microvessel density between the patients with the other *ADGRG6* enhancer C > T/G mutation (chr. 6: 142,706,209) and no mutations (Fig. 3c). To explore the association between *ADGRG6* enhancer alterations and individual survival, we performed Kaplan–Meier survival analysis on the additional UBC cohort and found that individuals with *ADGRG6* enhancer mutations had a much worse prognosis compared to those without mutation in both NMIBC and MIBC subcohorts (Fig. 3d). In addition, we found that SW780 and 5637 cells, which originate from UBCs and carry the *ADGRG6* enhancer mutation (chr. 6: 142,706,209; C > T or C > G transitions, respectively), expressed relatively higher level of *ADGRG6* than other UBC cells without this mutation (Supplementary Fig. 4a), and observed that depletion of *ADGRG6* expression in UBC cells compromised their abilities to recruit endothelial cells and induce tube formation (Fig. 3e, f). These results indicate that the mutation in *ADGRG6* enhancer changes its regulatory activity and acts like a novel oncogenic driver critical for pathological angiogenesis in UBC, providing a potential target for bladder cancer diagnostic screening and treatment.

Two hotspot mutations were also observed in the promoters of *PLEKHS1* and *LEPROTL1*, as well as the UTR of *TBC1D12* (Supplementary Fig. 5). Although recurrently promoter or UTR mutations for *PLEKHS1* and *TBC1D12* have been reported in several cancers, the functional role of these genes in tumorigenesis still remains uncharacterized^9,10^. *LEPROTL1* is a largely uncharacterized gene and has not previously been linked to tumorigenesis. In contrast to the hotspot mutations for *ADGRG6*, the noncoding regulatory element mutations in *WDR74* and *PLXDC1* were broadly distributed across numerous positions and seemed to occur as clusters of several mutations within the same sample (Supplementary Fig. 5). These hotspots in noncoding genomic regions displayed many base substitutions of mutational signature A that is significant enrichment of C > T and C > G mutations and may be induced by the activity of APOBEC enzyme^21^.

### Whole-genome copy-number alterations

We profiled the UBC tumors for somatic copy-number alterations (SCNAs), and observed that some chromosomal arms or entire chromosomes had undergone large-scale copy-number gain or loss. Significantly chromosomal arm-level changes included gains of 1q, 5p, 8q, 11q, 20p, and 20q and losses of 5q, 6q, 8p, 9p, 9q, 11p, 17p, 18q, and 21p (Fig. 1e and Supplementary Fig. 6). The overall somatic copy-number aberration pattern was broadly consistent with previously studies, and no significantly disparate pattern was found between non-muscle-invasive and muscle-invasive tumors. Profiling of SCNAs identified many putative UBC driver genes, which include well-known tumor suppressor genes and oncogenes (as listed in the COSMIC database^22^, e.g., *DDR2*, *PTPRB*, and *SYK*), as well as other putative SCNA drivers (Supplementary Fig. 7).

### Characterization of SVs and recurrently mutated genes

**Fig. 4:**
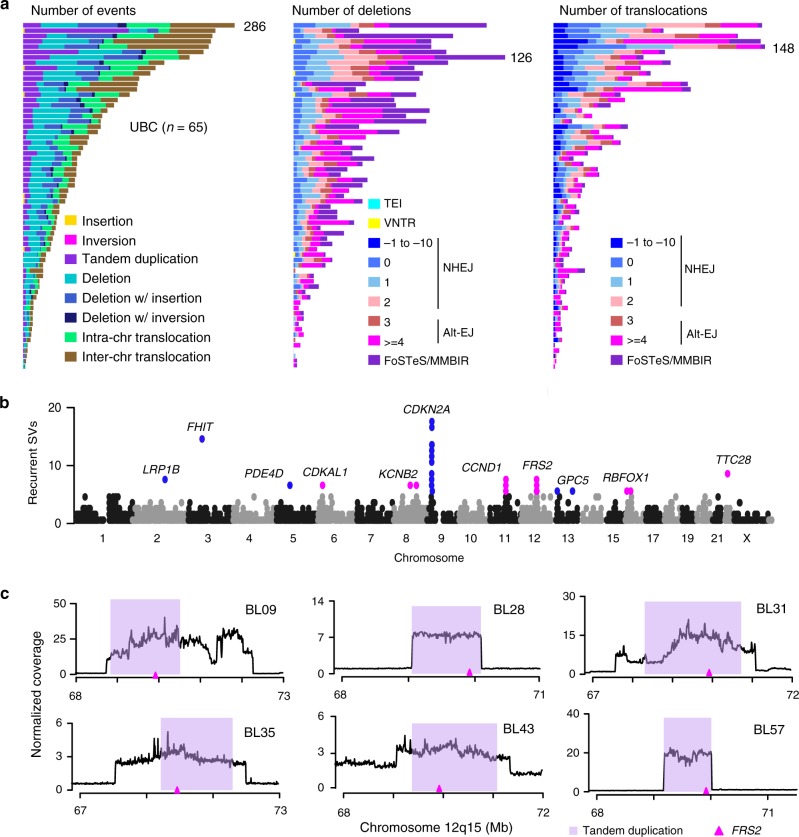
Analysis of somatic structural variations and SV-affected genes in UBC. **a** Frequencies of types of SVs (left) as well as underlying mechanisms for deletion (middle) and translocation (right) events across 65 UBC tumors. The colored bar charts display the number of events scaled by the maximum number of events (as noted) in each tumor. TEI transposable element insertion, VNTR variable number of tandem repeat, NHEJ nonhomologous end joining through 0–2 bp homology (0–2) or 1–10 bp insertion (−1 to −10) at breakpoint, alt-EJ alternative end joining through 3–100 bp homology (3, ≥4); FoSTeS/MMBIR, fork stalling and template switching or micro-homology mediated break-induced repair. **b** Summary of genes affected by SV breakpoints across the whole genome. Each dot represents a SV-affected gene which occurring in ≥5 tumors are shown in magenta (SVs mainly responsible for gene duplication) or blue (SVs mainly responsible for gene deletion), and significantly altered genes (in ≥10% of tumors) are annotated. **c** Normalized coverage of 12q15 region containing *FRS2* gene illustrates *FRS2* duplication in six UBCs. The purple ribbons represent the predicted tandem duplication regions, and the genomic location of *FRS2* was indicated by the magenta triangle

Larger scale SVs including insertions, inversions, tandem duplications, deletions, translocations, and complex rearrangements constitute another frequent type of normal gene functional alterations in tumorigenesis, and somatic SVs have been characterized in several cancers^8,23–25^. A total of 5391 somatic SVs were identified from the 65 UBC genomes, and the number of SVs was remarkable variation among individuals, ranging from 3 to 286 (Supplementary Data 1 and Data 2). Eight categories of SVs were observed, and the frequency of different types of SVs in each sample was displayed (Fig. 4a). In terms of SV types, translocations (39%) were the most abundant event type, whereas deletions and tandem duplications made up 28% and 16%, respectively. The mechanisms involved in formation of deletions and translocations were predicted as shown in Fig. 4a. The vast majority of somatic deletions were formed by alternative end joining (alt-EJ) and fork stalling and template switching or micro-homology mediated break-induced repair. For translocations, alt-EJ and nonhomologous end-joining were the dominant mechanisms, with alt-EJ being more abundant in most cases.

To examine how SVs affected UBC drivers, the occurrence of SVs within the region of coding genes was compared across samples (Fig. 4b). We found that 23 genes contained SV breakpoints in five or more tumors (Fig. 1f). Among these genes, *CCND1* and *MDM2* are known to be oncogenes and *CDKN2A* is a tumor suppressor. In addition, SVs are likely to occur in common fragile sites, and a cluster of fragile genes contained SV breakpoints in two or more samples (Supplementary Fig. 8). Recurrent SVs were also identified in *TP53*, *KRAS*, and *PIK3CA*, suggesting that important drivers might be affected by different mutational mechanisms in UBC (Supplementary Fig. 9).

**Fig. 5:**
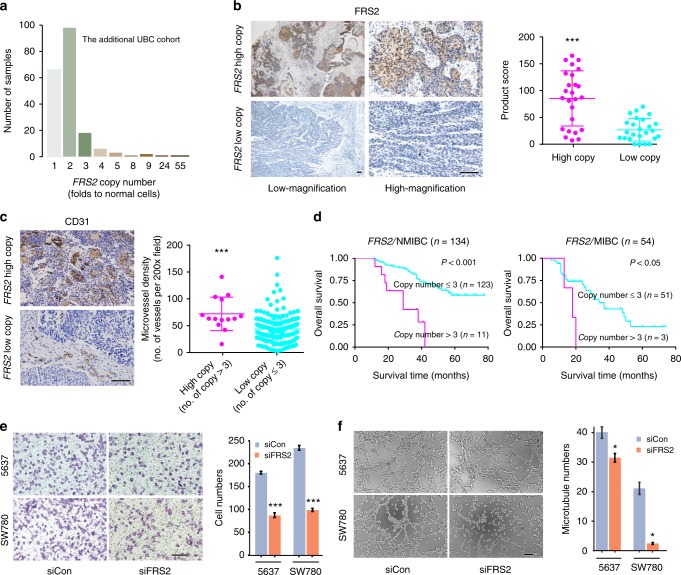
*FRS2* duplications predict poor prognosis and promoter tumor angiogenesis in UBC. **a** qPCR analysis in an additional 196 UBCs confirms the high-level duplication of *FRS2* in UBCs. **b** Immunohistochemical staining of 50 UBC tumors (25 tumors with *FRS2* high copy and others with *FRS2* low copy) displaying consequences of copy-number gain in *FRS2. FRS2* gene duplication leads to FRS2 protein overexpression in tumor tissues. **c** Immunohistochemical analysis of microvessel density in two different clusters of UBC tumors: one cluster with *FRS2* gene copy > 3 (*n* = 14) and the other cluster with *FRS2* gene copy ≤ 3 (*n* = 174). The microvessel densities were determined as described in methods. The three horizontal lines represent mean ± SD for the subjects. ***P < 0.001 vs. low-copy group, two-sided unpaired *t*-test with Welch’s correction (**b**) or Mann–Whitney test (**c**). **d** Kaplan–Meier survival curves for two clusters of patients (as mentioned in part **c**). The former cluster patients have poorer prognosis as compared with patients of the latter cluster in both nonmuscle-invasive bladder cancer (NMIBC) and muscle-invasive bladder cancer (MIBC) subcohorts. Statistical significance was determined by log-rank test. **e**, **f** Knockdown of FRS2 in 5637 and SW780 cells attenuated their abilities to recruit human umbilical vein endothelial cells (**e**) and induce tube formation (**f**). Scale bars, 100 µm. Error bars represent the SEM. The data shown represent averages from three independent experiments and were statistically analyzed by two-sided t test. *P < 0.05, **P < 0.01, ***P < 0.001 vs. siCon

Beside well-known cancer-associated genes, 6 UBCs had supporting SVs responsible for *FRS2* duplication with 3–25-fold increase of gene copy numbers (Fig. 4c). In addition, quantitative PCR (qPCR) analysis of the above additional UBC cohort further demonstrated the high-level amplification of *FRS2* in UBCs (Fig. 5a). Patient and mutation characteristics of the initial cohort of 65 UBCs and the additional cohort of 196 UBCs were summarized and compared (Supplementary Table 8). The duplication status of *FRS2* was uncorrelated with patient sex, age, and clinical phenotype in the additional UBC cohort (Supplementary Table 7). The results from immunohistochemistry staining of matched cases suggested that UBC tumors with *FRS2* duplication showed increased expression compared with those in other tumors without duplication (Fig. 5b). Analysis of the additional UBC cohort revealed that increased *FRS2* gene copy number was associated with an increased microvessel density and poor prognosis (Fig. 5c, d). Furthermore, univariate and multivariate Cox’s regression analyses were performed by integrating several risk factors including sex, age, histologic grade, clinical stage, surgical approach, *FRS2* duplication, and *ADGRG6* enhancer mutation. In the univariate analyses, *FRS2* duplication and *ADGRG6* enhancer mutation were found to be adverse prognostic factors for overall survival (Supplementary Table 9). Multivariate analysis revealed *FRS2* duplication (hazard ratio = 5.7; 95% confidence interval = 2.8–11.5; *P* < 0.001) and *ADGRG6* enhancer mutation (hazard ratio = 3.0; 95% confidence interval = 1.7–5.2; *P* < 0.001) to be independent prognostic factors for poor survival (Supplementary Table 9). FRS2 is a fibroblast growth factor receptor (FGFR)-associated protein required for signal transduction from activated FGFR, mediating numerous physiologic processes including cell proliferation, migration, and differentiation^26,27^. We then knocked down *FRS2* in 5637 and SW780 cells, which express relatively higher levels of *FRS2* (Supplementary Fig. 4b), and observed that *FRS2* silencing attenuated UBC cellular malignant phenotypes (cell proliferation), as well as their abilities to recruit endothelial cells and induce tube formation (Fig. 5e, f). Similarly, measuring the mRNA levels of both *FRS2* and *ADGRG6* in the siRNA knockdown experiments might demonstrate that the effects on recruitment and tube formation of endothelial cells directly resulted from the siRNA-mediated gene silencing (Supplementary Fig. 10). Nevertheless, knockdown of *ADGRG6* in 5637 cells to some extent inhibited *FRS2* expression, implying that the cell membrane protein ADGRG6 might directly or indirectly regulate FRS2. These results indicate a high frequency of *FRS2* amplification in UBC and uncover its angiogenic role in tumor development.

### Aberrant angiogenesis pathway in UBC

**Fig. 6:**
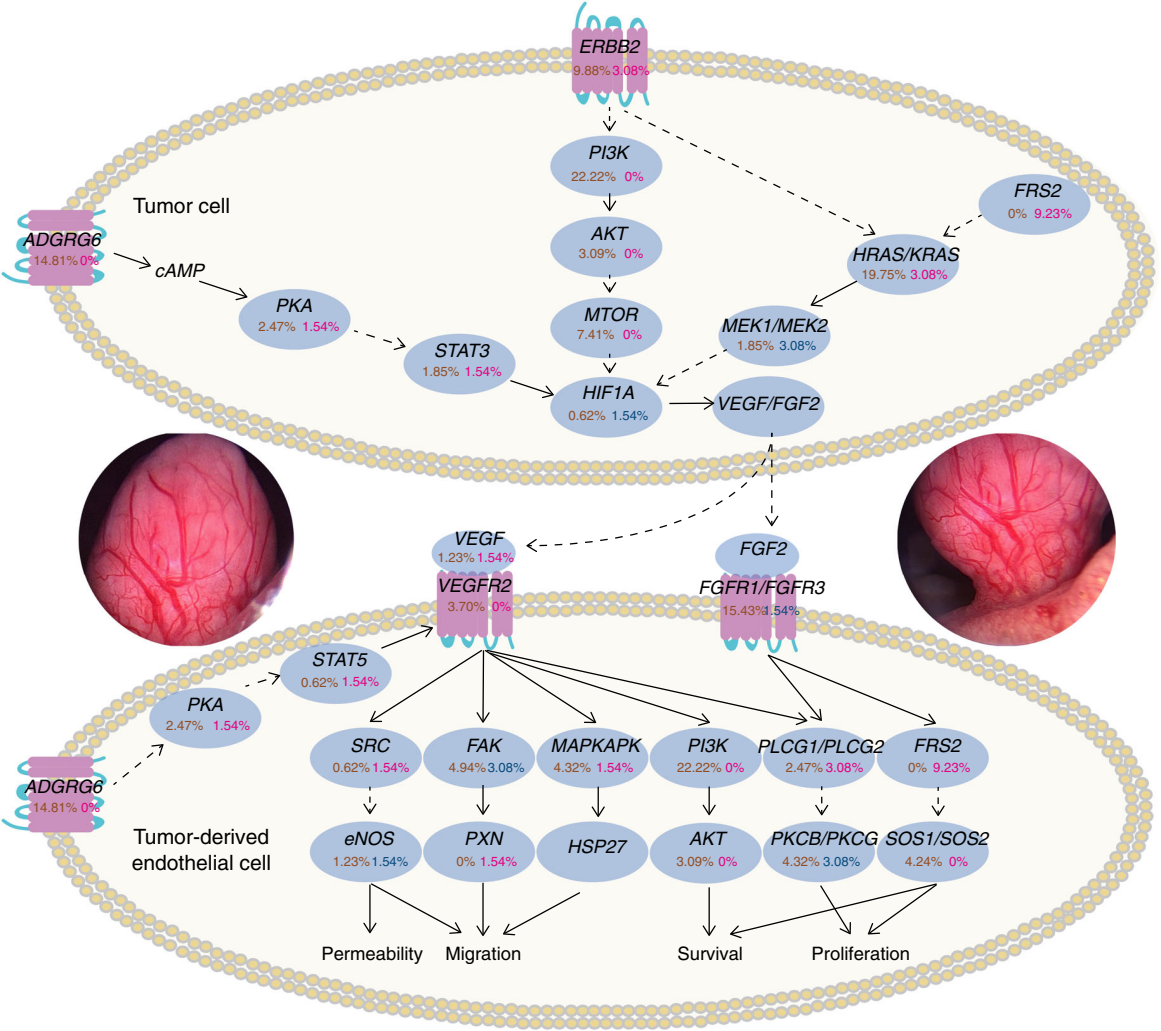
Frequent genetic alterations in genes from angiogenesis pathway in UBC. Alterations are defined as SNVs/indels and SVs (leading to the target gene amplification or deletion). Alteration frequencies are expressed as a percentage of analyzed cases (65 cases for SV; 164 cases for SNV/indel). SNV/indel is indicated in brown font, amplification in magenta font, and deletion in blue font. The middle insets show the high level of microvessel density within UBC tumors

The high level of microvessel density within UBC tumors is clearly displayed (Fig. 6 and Supplementary Movie 1). We integrated SNVs and CNAs from the 65 UBCs as well as SNVs from our previous 99 UBCs^4^ and determined genomic alterations of angiogenesis-related genes. Obviously, in addition to *ADGRG6* and *FRS2*, a set of genes involved in angiogenesis were frequently altered (Fig. 6). *HRAS/KRAS*, *PI3K*, *FGFR1/FGFR3*, *FAK*, *MTOR*, and *PKCB/PKCG*, these genes with important roles in angiogenesis, were altered in 23%, 22%, 17%, 8%, 7%, and 7% of the tumors, respectively. Discovery of high-frequency alterations in angiogenesis regulators could help to reveal molecular mechanisms for pathological angiogenesis in UBC tumorigenesis.

## Discussion

Angiogenesis is considered an independent prognostic indicator of many cancers. Vascular endothelial growth factor (VEGF) family and their receptors have been shown to be the fundamental regulators in the cell signaling of angiogenesis^28^. Other pathways, angiopoietin/Tie and hypoxia-inducible factor, etc. are also deeply involved in and cooperate with VEGF system to promote the angiogenic process in cancer development and progression^29^. It was recently reported that the ADGRG6 promotes mouse retinal angiogenesis and zebrafish embryonic vascular development by modulating VEGFR2 expression through STAT5-mediated transcription^20^. Activation of FGFR signaling pathway as a result of *FRS2* adapter amplification was recently identified in high-grade serous ovarian cancer and liposarcoma^26,30^. In addition, hyperactivation of the FRS2-mediated signals promoted tumor angiogenesis and predicted poor outcomes in prostate carcinoma patients^31^. In this study, whole-genome and targeted sequencing of UBCs identified recurrent *ADGRG6* enhancer mutations and *FRS2* amplifications, as well as high-frequency alterations in a group of angiogenesis regulators, which may significantly facilitate our understanding of molecular mechanisms underlying pathological angiogenesis in the highly vascularized cancer.

Structural variant analysis has the potential to become a new classification method for delineating more specific tumor subtypes. Based on structural variation profiles, pancreatic cancer was classified into different subtypes with potential clinical relevance^25^. Although focused molecular analyses have identified clinically relevant subtypes of UBC^2,32^, UBC subtyping using structural rearrangements may significantly improve its clinical management. The distribution of SV events was used to classify UBCs into the following four subtypes. Forty-eight percent of UBC genomes containing less than 50 SVs were classified as stable subtype (Supplementary Fig. 11). UBCs exhibiting a significant focal SV event on a few chromosomes or a moderate range of non-random chromosomal damage with less than 200 SV events were classified as locally rearranged or scattered subtype, respectively (Supplementary Fig. 12 and Fig. 13). The remaining UBCs stating a large-scale of genomic instability with more than 200 SV events were classified as unstable subtype (Supplementary Fig. 14).

The underlying mechanisms of oncogenic events driven by SVs were further explored. Many UBC patients (45% of all samples) harbor clusters of localized hypermutation in the genome, a process termed kataegis likely resulting from APOBEC-mediated mutagenesis with enrichment of C > T and C > G alterations^21,24^, and a series of genes involving in tumorigenesis were affected by the process (Fig. 1g, Supplementary Table 10 and Supplementary Fig. 15). In parallel, another mutation mechanism, chromothripsis characterized by extensive transitions oscillating between two or three copy-number states in the affected chromosomes^33^, has been identified in eight UBCs (Fig. 1g). In addition to general transition, *CASC15*, a tumor suppressor long intergenic noncoding RNA at 6p22^34^, was disturbed by the chromothripsis-associated rearrangements and gene fusion events involving *CASC15* were identified in five UBCs (Supplementary Data 3 and Supplementary Fig. 16). Other fusion events across 65 UBC genomes were also screened (Supplementary Data 3). The breakage-fusion-bridge (BFB) cycle could generate variability in chromosome structure characterized with telomere loss and fold-back inversion, and it is also a well-established mechanism of tumor genome instability^35^. BFB events were identified in five UBC genomes (Fig. 1g and Supplementary Table 11). Notably, in UBC-BL05 and UBC-BL02, the BFB-associated rearrangements led to the amplification of *PTPRT*, which encodes a receptor protein tyrosine phosphatase and plays a vital role in tumor development^36^ (Supplementary Fig. 17). Together, these findings demonstrated diverse types of SVs contributing to the UBC mutational landscape and the implicated complex genomic rearrangements as an integral part of mutation mechanisms in UBC carcinogenesis.

In summary, this study provides the most comprehensive description, to date, of genetic alterations in UBC and facilitates discovery of a greatly extended genetic profile as well as multiple previously unreported oncogenetic mechanisms of UBC tumorigenesis. Our analyses emphasize the important tumorigenic mutations occurring in noncoding regions and diverse models of SVs contributing to genomic damage in this tumor. In addition, the proof of concept data presented in this study suggests that genetic alterations and aberrant expression of angiogenesis-related genes have potential implications for the selection of targeted therapies in the care of UBC patients.

## Methods

### Samples description and data processing

A cohort of 65 fresh-frozen tumor tissues and matched peripheral blood samples from patients who newly diagnosed with UBC at the member institutions of the Urinogenital Cancer Genomics Consortium in China (UCGC) was subjected to whole-genome sequencing. These patients were treated with surgical resection by either transurethral resection or radical cystectomy, and none of them had received prior systemic and intravesical chemotherapy or radiotherapy. Raw high-throughput data were filtered with SOAPnuke (v. 1.5) to remove sequence adapters and low-quality reads. Then, the high-quality clean reads were mapped to the human reference genome (hg19, NCBI build GRCh37) with Burrows–Wheeler Aligner (BWA, v. 0.7.12)^37^ and processed with Picard (v. 1.127) to mark duplicate reads. Genome Analysis Toolkit (v. 3.2)^38^ was used to complete local realignment and improve alignment accuracy. Point mutations and indels were detected using Mutect (v. 1.1.4)^39^ and Strelka (v. 1.0.15)^40^ software, respectively. Copy-number variations and structure variations were identified by Segseq (v. 1.01)^41^ and Meerkat (v. 0.185)^23^ software using the default settings. The target mutations were verified by inspecting IGV^42^ and checking the support read numbers in the BAM files. Ethics board approval was obtained at all institutions (the Ethics Committee of Shenzhen Luohu People’s Hospital and the Ethics Committee of Capital Medical University Affiliated Beijing Chaoyang Hospital) for patient recruitment and each participant in this study was properly informed following the guidelines of the institutional ethics review boards.

### Analysis of mutation patterns and signatures

There are 96 possible mutation types occurring in a trinucleotide context (C:G > A:T, C:G > G:C, C:G > T:A, T:A > A:T, T:A > C:G, and T:A > G:C with the bases immediately 5′ and 3′ to each substitution). Based on the trinucleotide context mutation frequency spectrum, different mutational processes generated diverse combinations of mutation types which were termed as “Mutational Signatures”. The nonnegative matrix factorization (NMF) algorithm was proposed as a new method for deciphering those mutational signatures. According to the description of R package named Somatic Signatures^43^, the NMF run was iterated until convergence or until 1000 iterations were performed. We tried a series of NMF runs with various numbers of signatures from 2 to 15, and selected four as the best number because of the signature stability and reconstruction error achieving the optimal balance (Supplementary Fig. 2). After getting the validated mutational signatures, we compared these signatures with COSMIC signature database to measure the cosine similarity distance.

### Mutations annotation in noncoding region

Somatic mutations were analyzed and annotated in noncoding regions which were defined using Funseq2 package (Funseq 2.1.2)^44^. The promoter regions were annotated according to PCAWG consortium’s definition, and the enhancer regions were identified by correlating histone modifications with gene-expression data. The intron and UTR region was defined by GENCODE database^45^. The transcription factor binding motifs were defined by ENCODE database^18^ and Roadmap Epigenomics project^46^.

### Testing mutation frequencies of regulatory regions

The mutation frequencies of regulatory regions were tested as previously described by Weinhold et al.^9^ with slight modifications. Briefly, the whole genome was divided into numerous coterminous 1 kb regions, and the number of SNVs or SV breakpoints in each region was compared to that in 1 Mb flanking region (local approach, 500 kb upstream and 500 kb downstream) or whole-genome background region (global approach). All effective background regions in both approaches were mapped at high depth (tumor > 14×, normal > 8×). The mutation frequency of each region was estimated by dividing the total number of somatic mutations by the effective length of the background region. *P* values were computed and adjusted using the method described by Weinhold et al.^9^. All mutated regulatory regions reaching statistical significant (adjusted *P* < 0.05) in both approaches were annotated as promoter, intron, UTR and enhancer using Funseq2 package, excepting excluded blacklist regions from ENCODE project and the 263 public SGDP samples across 128 diverse populations^47^.

### Detection of SVs

Identification of SVs from whole-genome sequencing data was performed using the Meerkat package as previously described by Yang et al.^23^. In short, we aligned all sequencing reads against the human reference genome (hg19) to obtain the soft-clipped and unmapped reads, and identified discordant read pairs by re-mapping these reads back to the reference genome. Then we predicted breakpoint junctions from supported reads and redefined precise breakpoints by local alignments. The SVs-formed mechanisms were determined based on homology and breakpoints features. Somatic SVs were obtained by filtering out germline SVs and low false positive events, and only high confidence calls were used for further analysis.

### Classification of UBC subtypes

Based on the pattern of structural rearrangements, four UBC subtypes were classified as previously described in pancreatic cancer^25^. The rules used to determine these subtypes were summarized briefly as follows: (1) stable tumors harbor less than 50 structural rearrangements located randomly across the genome; (2) locally rearranged tumors contain at least 50 somatic events with more than 25% of these events enriched in one chromosome; (3) Scattered tumors contain 50–200 structural rearrangements scattered throughout the genome; (4) unstable tumors are massively rearranged with more than 200 structural rearrangements generally scattered across the genome.

### Identification of kataegis

The genomic regions containing kataegis were identified as follows^24^: (1) containing six or more consecutive mutations with an intermutation spacing of less than or equal to 1 kb; (2) the mutation rate for targeted region significantly higher than those for neighboring 50 kb scopes on each side and the whole-genome region; and (3) surrounded by SV breakpoints.

### Detection of BFB

We defined BFB events according to the four stringent standards proposed by Zakov et al.^35^: (1) fold-back inversion detected by Meerkat; (2) the two ends of breakpoints of fold-back inversion must be spaced less than 20 kb apart; (3) the fold-back inversion region with significant copy-number change; and (4) at least twofold-back inversions located adjacent to the telomere.

### Inference of chromothripsis

To infer chromothripsis in UBCs, we adapted the criteria proposed by Korbel et al.^33^ The suspect chromothriptic regions as well as their chromothripsis scores were processed and produced by ShatterProof^48^ with the default hallmark weightings. The final score above 0.6 was considered as the occurrence of chromothripsis.

### Cell lines and cell culture

An immortalized normal uroepithelial cell line SV-HUC-1, human umbilical vein endothelial cells (HUVECs), and six UBC cell lines (5637, SW780, UM-UC-3, T24, TCCSUP, and RT4) were purchased from American Type Culture Collection. All cells were detected and found to be free of mycoplasma infection. These cells were grown and maintained in DMEM/F12 (SV-HUC-1), RPMI-1640 (5637, T24, and TCCSUP), DMEM (SW780 and UM-UC-3), McCoy’s 5a (RT4) or extracellular matrix (ECM) (HUVECs) medium supplemented with 10% fetal bovine serum at 37 ℃ in 5% CO_2_. Endogenous products encoded by target genes of interest were detected through quantitative reverse transcription PCR (RT-PCR) and/or immunoblotting analyses. For functional analysis, gene knockdown experiments were performed in the UBC cell lines with high endogenous expression.

### Cell transfection and conditioned medium collection

siRNA oligonucleotides against human *ADGRG6* (siADGRG6#1 and siADGRG6#2), *FRS2* (siFRS2), and a nontargeting negative control siRNA (siCon) were synthesized by Shanghai Sangon Biotechnology Co. Ltd. (Supplementary Table 12). Totally, 5637 and SW780 cells were plated into six-well plates, and mixtures of siRNA and Lipofectamine RNAiMAX reagent (Invitrogen) were added to each well as 30 nM siRNA solutions. After being transfected with siRNA for 24 h, the culture media were changed to 0.2% FBS RPMI-1640 or DMEM, and then conditioned mediums were collected after another 24 h.

### HUVEC tube formation and endothelial recruitment

HUVECs (1 × 10^4^) were seeded in 96-well plates coated with Matrigel (10 mg/ml) and cultured in the ECM medium with supplementation of the indicated conditioned medium (1:2) for 4–6 h at 37 °C. Images were acquired under a phase-contrast microscope and the tube numbers were counted in three individual wells. The endothelial recruitment assay was performed in transwell (24-well plates) inserts (Corning) with 8.0 µm pore polycarbonate membrane. 5637 and SW780 cells (8 × 10^4^/well) seeded in the lower chambers were transfected with the indicated siRNAs. After incubation for 36 h, the culture media were replaced with 0.2% FBS ECM, and the upper chambers were then seeded with 6 × 10^4^ serum-starved HUVECs in 50 µl 0.2% FBS ECM medium. After the co-culture for 30 h at 37 °C, HUVEC cells that migrated to the lower surface of membranes were stained and counted under a light microscope in five fields/wells. At least three independent experiments were performed.

### Quantitative RT-PCR and western blotting analysis

Total RNA was extracted using the EZNA® Total RNA Kit (Omega Bio-tek) and subjected to cDNA synthesis using PrimeScript RT reagent Kit (Takara). Quantitative RT-PCR was performed with TransStart Tip Green qPCR SuperMix (TransGen), using primers for *ZFP36L1*, *ELF3*, *ADGRG6*, *FRS2*, or *GAPDH* (Supplementary Table 12). The relative expression of target genes was determined by normalized to that for *GAPDH*. All reactions were done in triplicate. Cells were lysed in RIPA buffer supplemented with protease inhibitor mixture, and lysates were cleared by centrifugation at 12,000*g* for 15 min. The protein concentration of each sample was determined by the Bradford method. Protein extracts were separated by sodium dodecyl sulfate polyacrylamide gel electrophoresis and electroblotted onto polyvinylidene difluoride membranes. The following antibodies were used: anti-ADGRG6 (Abcam, catalog no. ab75356); anti-FRS2 (R&D Systems, catalog no. MAB4069); and anti-GAPDH (Cell Signaling, catalog no. 2118 S). Membranes were probed with secondary anti-rabbit or mouse horseradish peroxidase-labeled antibodies (CST, catalog no. 7074S and 7076S), and the antigen–antibody reaction was visualized by chemiluminescence. Equal protein loading was confirmed with antibodies against GAPDH.

### Sanger sequencing and qPCR

An additional independent cohort consisted of 196 UBC patients from UCGC. These patients were properly informed before recruitment for tumor genetic analysis and clinical research under a protocol that was approved by the institutional ethics review boards of all participating centers (the Ethics Committee of Shenzhen Luohu People’s Hospital and the Ethics Committee of Capital Medical University Affiliated Beijing Chaoyang Hospital). All of the selected UBC patients had not received prior systemic and intravesical chemotherapy or radiotherapy, and their formalin-fixed paraffin-embedded (FFPE) tumor tissues, clinical features as well as follow-up information were well saved in the UCGC Biospecimens Bank. Patient regular follow-up evaluation consisted of physical examination, cytology, ultrasound, and/or cystoscopy. The initial follow-up was organized at 3 months postoperatively. Then patients were followed up at 3-month intervals the first year, 6-month intervals the second year, and annually thereafter. Radiographic evaluation of the urinary diversion and chest radiography were performed at 3 months after operation, then every 2 years thereafter unless otherwise clinically indicated. Elective abdominal/pelvic computerized tomography scans and bone scans were performed only if clinically indicated. If patients decided to discontinue their follow-up participation, they were contacted by telephone to maintain a strict follow-up. Overall survival was defined as the time from surgery to the date of death or last follow-up, and was estimated using Kaplan–Meier analysis with the log-rank test for comparison of groups. Univariate and multivariate analyses with Cox’s proportional hazards regression model were used to assess the impact of clinical variables on patient survival. The gDNAs of the additional UBC cohort were isolated from FFPE tissue sections using the TIANamp FFPE DNA Kit (Tiangen Biotech) according to the user manual. The gDNAs of the above cell lines were also extracted. To characterize the mutations of *ADGRG6* enhancer and *FRS2* amplification in UBCs and bladder cancer cell lines, primer pairs for *ADGRG6*, *FRS2*, and reference gene *GAPDH* were designed (Supplementary Table 12) for Sanger sequencing and qPCR analysis. Sanger sequencing was conducted using an automated ABI sequencer, and qPCR analysis was performed on the QuantStudio Dx instrument (Life Technologies). Relative gene quantification method was applied to calculate the fold change of *FRS2* copy number in UBCs to gDNA extracted from the normal blood or urine samples.

### Immunohistochemistry and microvessel density analyses

Based on the Sanger sequencing peak map of *ADGRG6* enhancer, the top 25 UBC tumors only with *ADGRG6* enhancer G > A mutation, the top 25 UBC tumors only with *ADGRG6* enhancer C > T mutation, and the random selection of 25 UBC tumors without *ADRGR6* enhancer mutations were subjected to immunohistochemistry analysis of ADGRG6. According to the copy number of *FRS2* in the tumor tissue section, the top 25 UBC tumors with *FRS2* high copy and the top 25 UBC tumors with *FRS2* low copy were subjected to immunohistochemistry analysis of FRS2. Immunohistochemistry was performed with standard protocol^49^ with specific antibodies as follows: anti-FRS2 (Abcam, catalog no. ab150058); anti-ADGRG6 (Abcam, catalog no. ab117092); and anti-CD31 (Abcam, catalog no. ab28364). In brief, after deparaffinized, rehydrated, and antigen retrieval, sections were incubated with special antibody at an ideal dilution, and subsequently stained with the DAB detection kit (Maixin). Slides were counterstained with hematoxylin. The overall product immunoreactive score for the detected protein was calculated for each case by multiplying the staining percentage (0–100%) with the numerical score of the staining intensity (none = 1, weak = 2, moderate = 3, strong = 4). Tumor-associated angiogenesis was assessed by the microvessel density according to the method described by Weidner et al.^50^ with minor modifications. Briefly, the anti-CD31 antibody was used to identify endothelial cells. Microvessel counts were evaluated on a 200x power microscopic field within the neovascular hotspot which was designated after scanning the entire section at 40× power. Any stained endothelial cell isolated from adjacent microvessels and other connective tissue elements was considered to represent a single microvessel. Counts were performed using digital imaging software (Image-Pro Plus 6.0) with three neovascular hotspots analyzed per case.

### Statistics in the experiments

Statistical analyses were conducted with SPSS (Version 22) or GraphPad Prism (Version 7.00) software. Data were derived from at least three independent experiments and shown as mean ± SEM. *P* value less than 0.05 was considered statistically significant.

## Supplementary information

Supplementary Information

Description of Additional Supplementary Files

Supplementary Data 1

Supplementary Data 2

Supplementary Data 3

Supplementary Movie 1

## Data Availability

The sequencing data of this study have been deposited in the European Genome-phenome Archive (EGA, https://ega-archive.org) at the EMBL-European Bioinformatics Institute (accession number: study, EGAS00001003388; dataset, EGAD00001004545). The data sets of previous reported 99 UBC cases are available in the Sequence Read Archive (SRA, https://www.ncbi.nlm.nih.gov/sra) under accession SRA063495. All the other relevant data are contained within the article or Supplementary files, or available from the corresponding author upon reasonable request.
