# Supplementary material for: Whole-genome sequencing identifies ADGRG6 enhancer mutations and FRS2 duplications as angiogenesis-related drivers in bladder cancer
Source: Nat Commun. 2019 Feb 12;10:720. doi: 10.1038/s41467-019-08576-5 (PMC6372626; doi:10.1038/s41467-019-08576-5)
Supplement: Supplementary file 1 — Supplementary Information [file 41467_2019_8576_MOESM1_ESM.doc]

**Supplementary Information**

**Whole-genome sequencing identifies *ADGRG6* enhancer mutations and *FRS2* duplications** **as angiogenesis-related drivers in bladder cancer**

**Wu et al.**

**Supplementary Figures**

**Supplementary Figure 1**

**a**

**b**


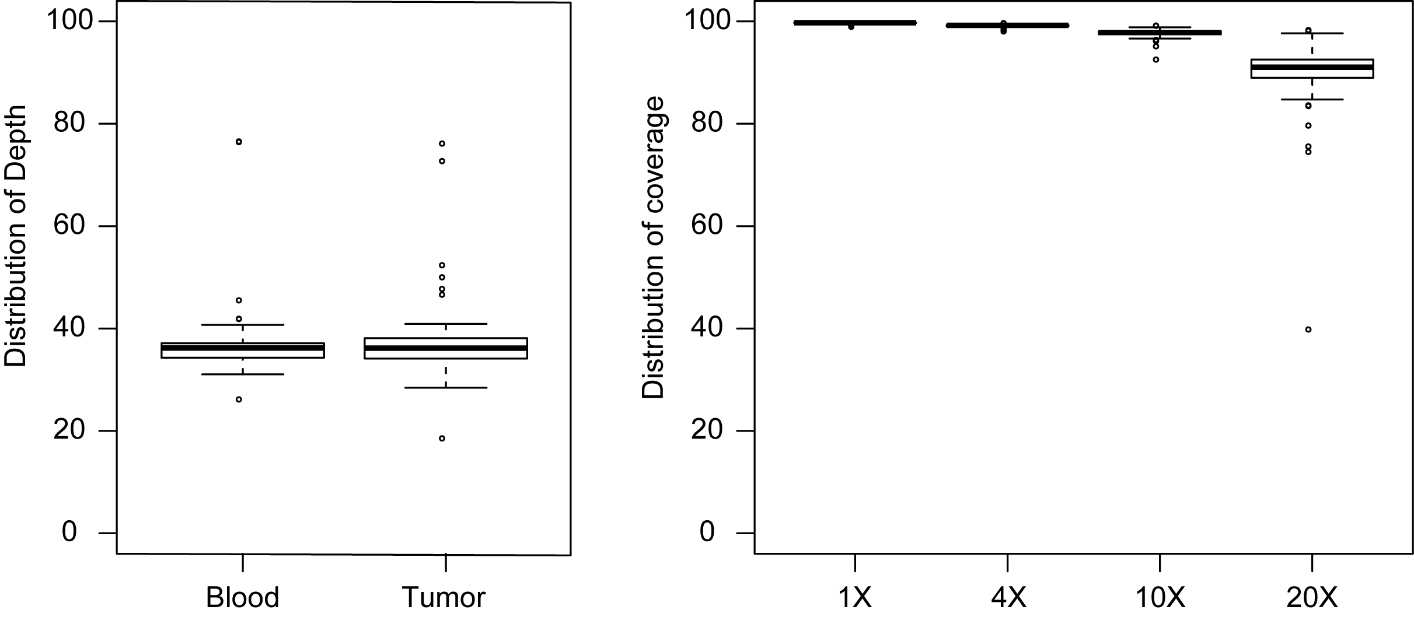


**Supplementary Figure 1. Distribution of depth and genome coverage for the peripheral blood (Blood) and tumor (Tumor) samples from 65 UBC patients analyzed by WGS.** **a** The box plot shows the distribution of mean coverage of all whole-genome sequencing samples. Boxes show the interquartile range (IQR) between first and third quartiles, and lines inside the boxes show the medians. Whiskers represent the highest and lowest values within 1.5 × IQR from the upper and lower quartiles, respectively. Outlier data are shown as points. **b** The box plot shows the distribution of fraction of whole-genome bases covered by at least 1X, 4X, 10 X and 20 X.

**Supplementary Figure 2**


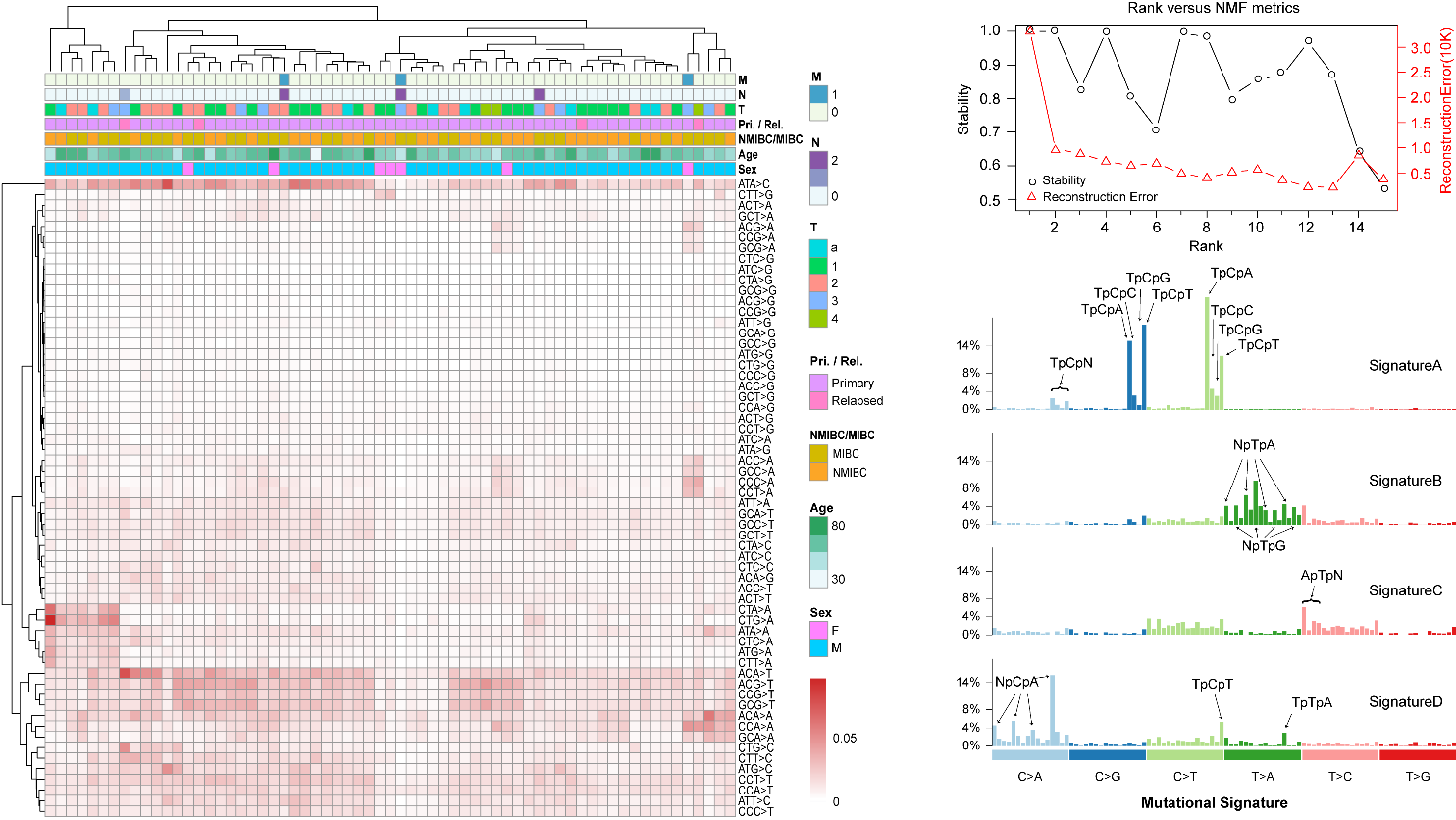


**a**

**b**

**c**

**Supplementary Figure 2. Identification of four mutational signatures in the UBC cohort.** **a** The frequency of the 96-substitution pattern of SNV in 65 UBC cases. The percent of each 96-substitution type is calculated and drawn by gradient red color with 0-1 value representing different transparency. The top legend shows the clinic information of each sample including T/N/M, primary/relapsed, superficial/invasive, age and sex. The X axis represents the cases, and the Y axis indicates the 96 substitution patterns. Each cluster is grouped with NMF method. **b** Stability and reconstruction error of non-negative-matrix factorization (NMF) analysis estimate four NMF metric was the best model because of the comparative balance with the signature high stability and the low reconstruction error. **c** The four mutational signatures identified in this UBC cohort, which were normalized by background tri-nucleotide frequencies.

**Supplementary Figure 3**


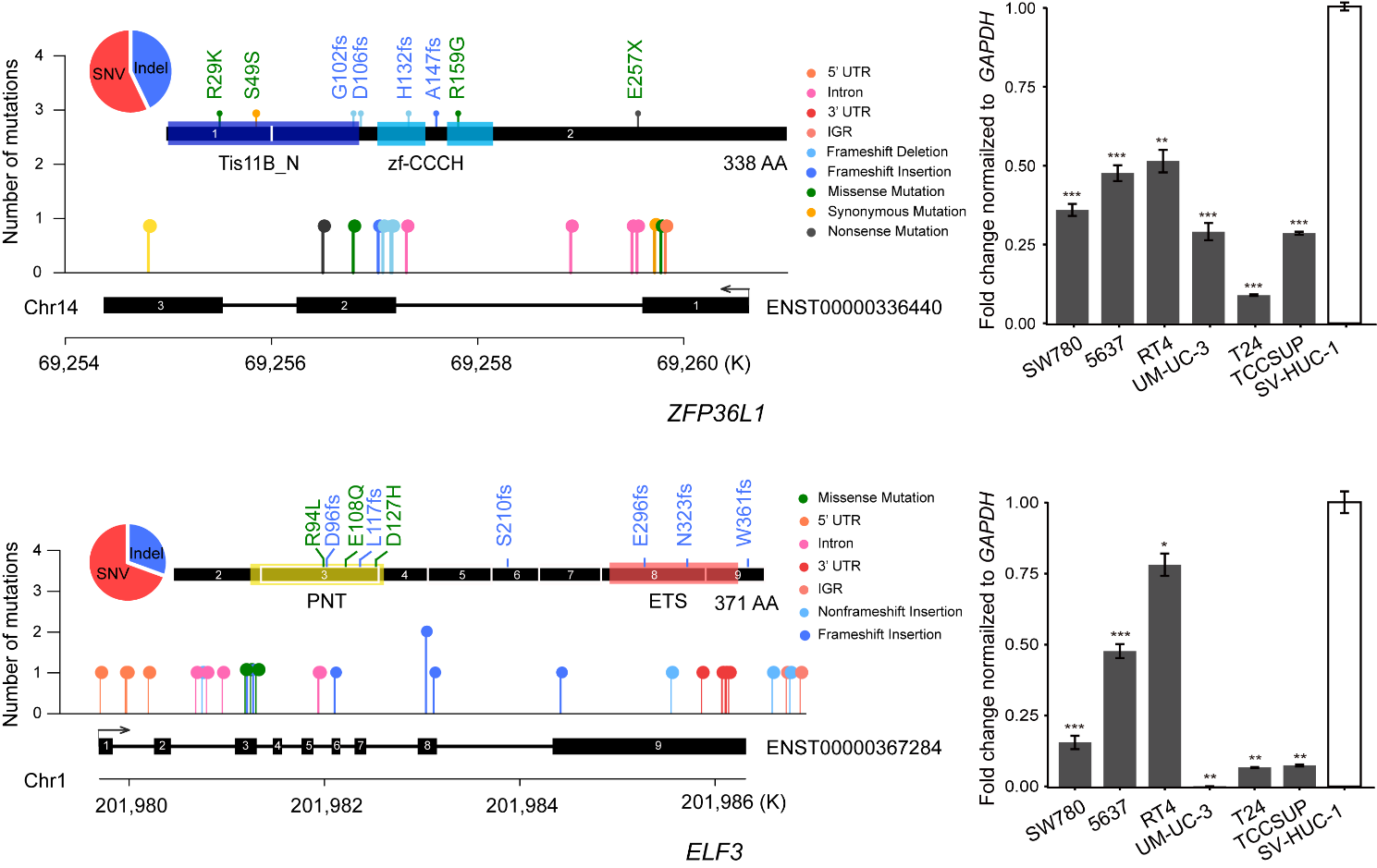


**a**

**b**

**Supplementary Figure 3. *ZFP36L1* and *ELF3* somatic mutations in UBC and their expressions in UBC cells. a** In the left panel, the x-axis represents SNVs and indels in *ZFP36L1*, and their proportions are displayed in the pie chart. Each point denotes one mutation, and the labeled colors depicts different mutation types which shown as in the legend. The exons are indicated below the point mutations, and the encoded protein and its alterations are illustrated above the x-axis. The mainly mutations are focused on the Tis11B_N and zf-CCCH domains. The *ZFP36L1* levels in 6 UBC cell lines and an immortalized normal bladder urothelial cell line (SV-HUC-1) were analysed by quantitative RT-PCR (right panel). **b** The detail mutation information of *ELF3* (left panel) and its expressions in UBC cells (right panel). The mainly mutations are focused on the PNT and ETS domains. The data shown represent averages from three independent experiments and were statistically analysed by two-sided t test. Error bars represent the SEM. * *P* < 0.05, ** *P* < 0.01, *** *P* < 0.001 versus SV-HUC-1.

**Supplementary Figure 4**


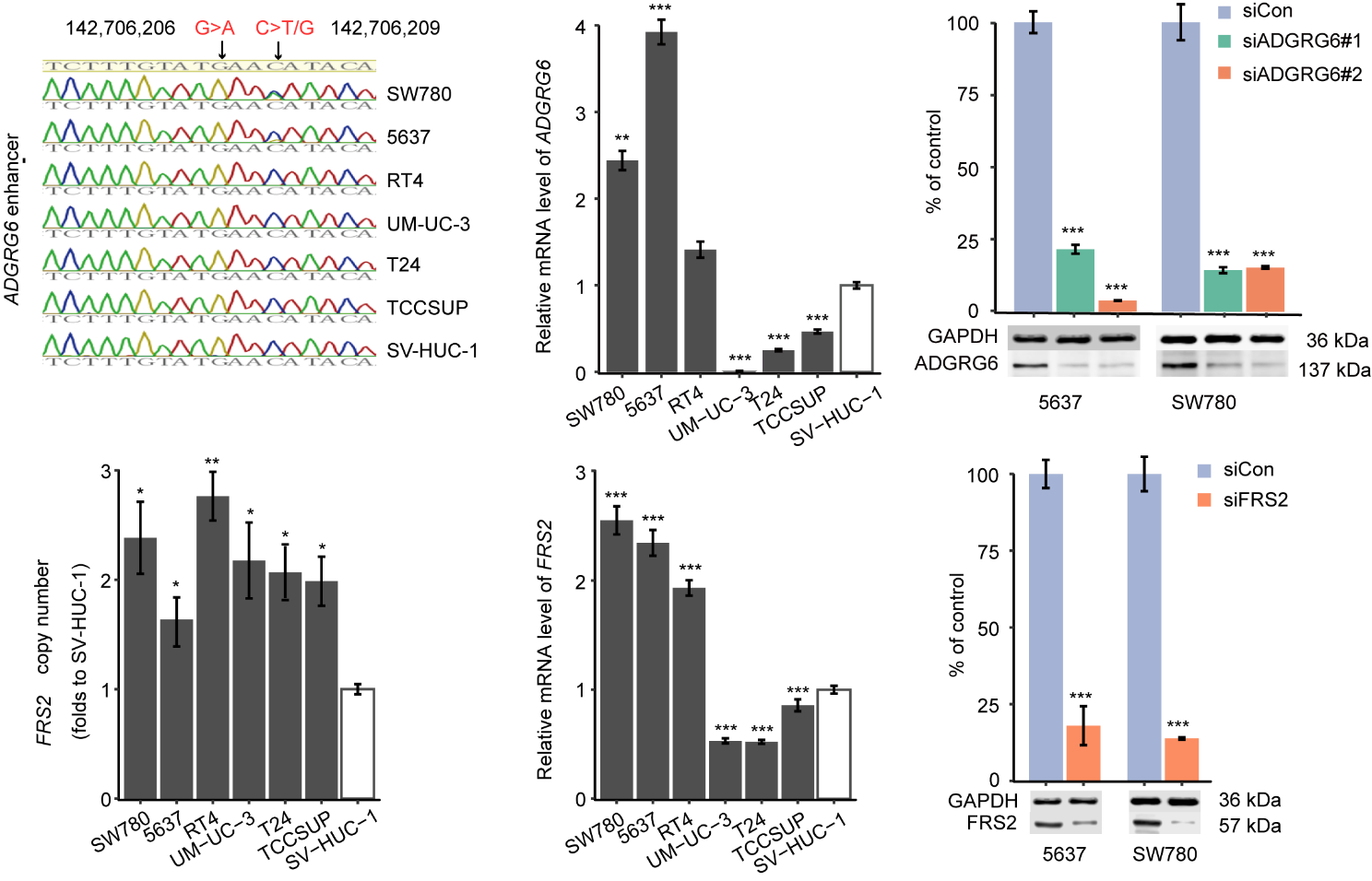


**a**

**b**

**Supplementary Figure 4. *ADGRG6* enhancer mutations and *FRS2* copy number variations in UBC cells. a** *ADGRG6* enhancer mutations (left) and expression levels (middle) in 6 UBC cell lines and an immortalized normal bladder urothelial cell line (SV-HUC-1) were analysed by Sanger sequencing and qPCR, respectively. 5637 and SW780 cells which harbor *ADGRG6* enhancer mutation express relatively higher level of *ADGRG6* than other UBC cells without the mutation. Knockdown of ADGRG6 by siRNA oligonucleotides in 5637 and SW780 cells (right) were confirmed by qPCR and western blotting. **b** FRS2 copy number (left) and expression level (middle) in 6 UBC cell lines and an immortalized normal bladder urothelial cell line (SV-HUC-1) were analysed by qPCR. Knockdown of FRS2 by siRNA oligonucleotides in 5637 and SW780 cells (right) were confirmed by qPCR and western blotting. The data shown represent averages from three independent experiments and were statistically analysed by two-sided t test. Error bars represent the SEM. * *P* < 0.05, ** *P* < 0.01, *** *P* < 0.001 versus SV-HUC-1.

**Supplementary Figure 5**


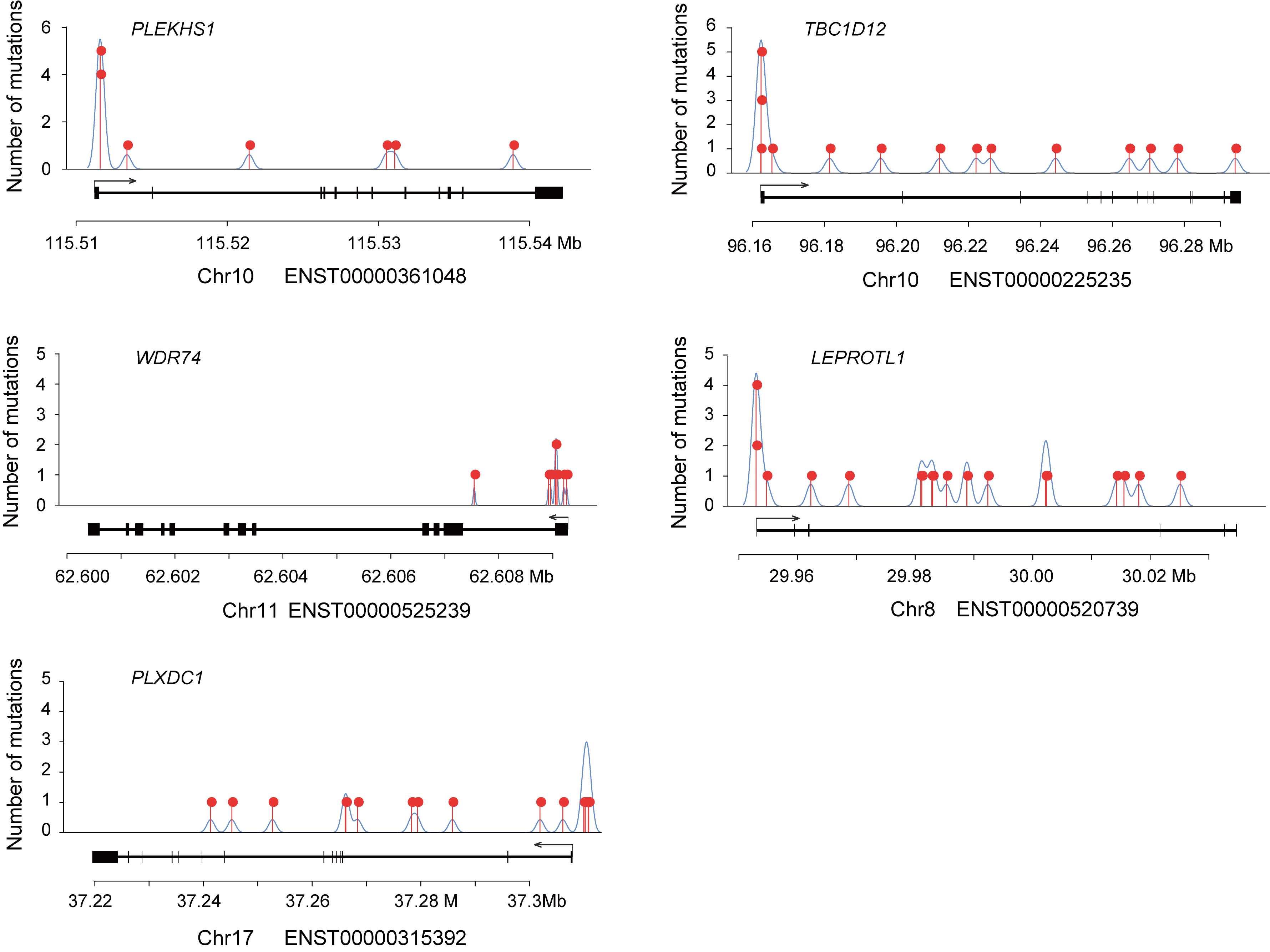


**a**

**b**

**c**

**d**

**e**

**Supplementary Figure 5. Frequent genes with noncoding regulatory element mutations.** The diagrams show the somatic mutations in *PLEKHS1* (a)*, TBC1D12* (b)*,* *WDR74* (c)*, LEPROTL1* (d) and *PLXDC1* (e)*,* as well as their noncoding regulatory elements. Mutation density across the region is shown as a light blue curve. The used transcription is labeled below the x axis.

**Supplementary Figure 6**


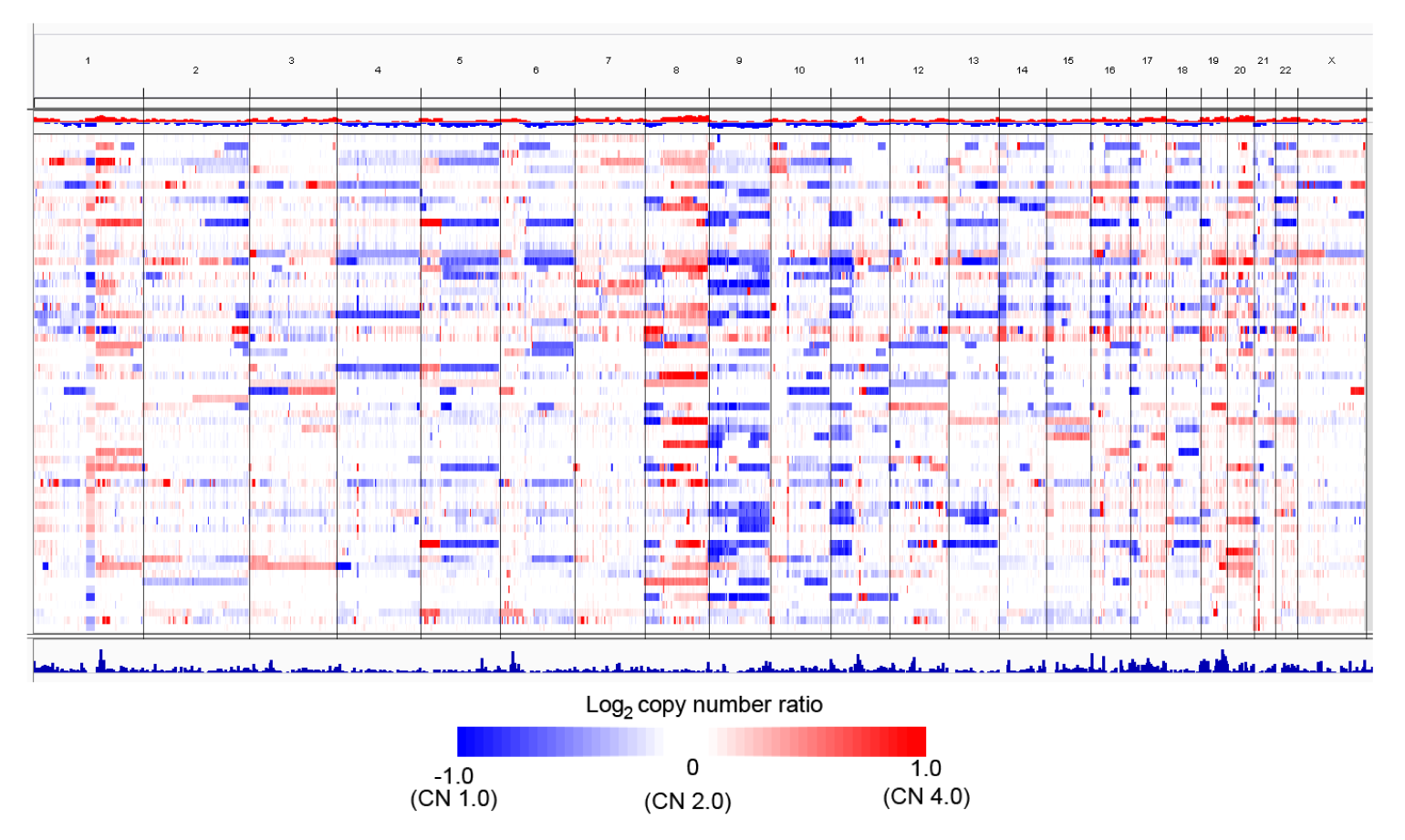


**Supplementary Figure 6. The landscape of copy number alteration in UBC cases displayed by Integrative Genomics Viewer (IGV).** The red line means the amplication, and the blue line means the deletion. The degrees of alteration are shown as gradient color, with red representing the maximum and blue representing the minimum**.**

.

**Supplementary Figure 7**


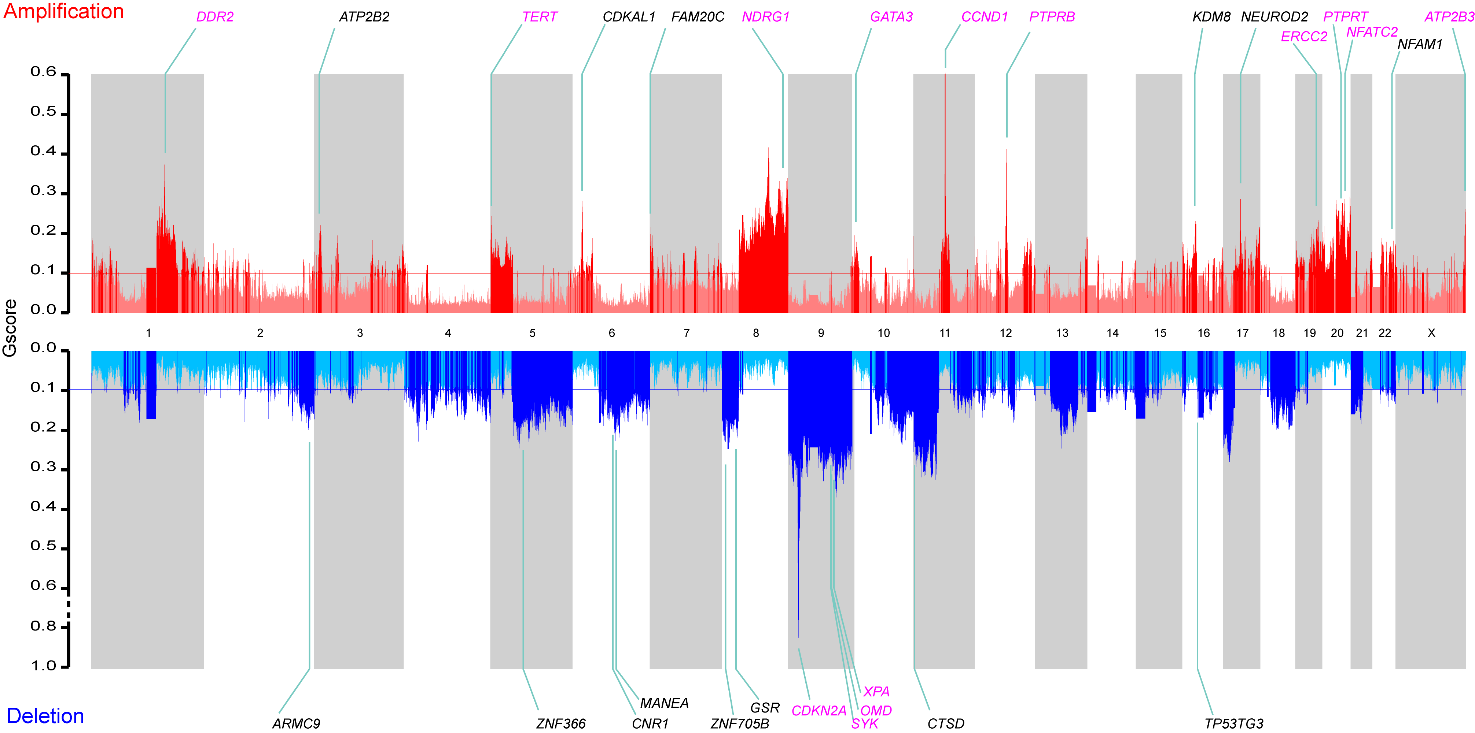


**Supplementary Figure 7. Overviews of affected chromosomal arms and genes.** The labeled genes locating at the peak of chromosome are putative cancer driver genes. The genes in magenta have been reported in the COSMIC database. The G-Score value of 0.098 are given as the horizontal solid lines, corresponding to the false discovery rate*q* value < 0.001.

**Supplementary Figure 8**


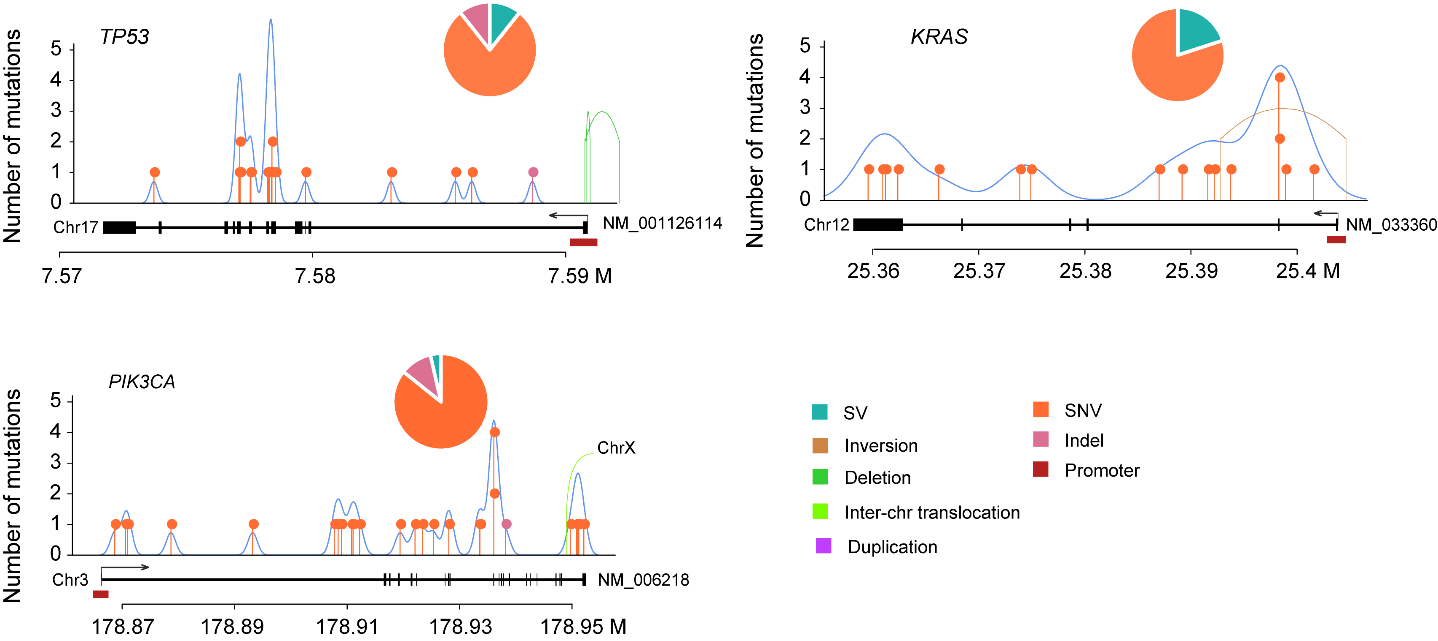


**c**

**a**

**b**

**Supplementary Figure 8. The schematic diagrams show the mutation structure of *TP53* (a)*, KRAS* (b) and *PIK3CA* (c).** The mutations including SNV/indel/SV are shown as red/pink/cyan color respectively, and each part’s proportion is displayed at pie chart. The detail types of SV are labeled as legend, and promoter regions which come from Encode database are drawn as brown.

**Supplementary Figure 9**


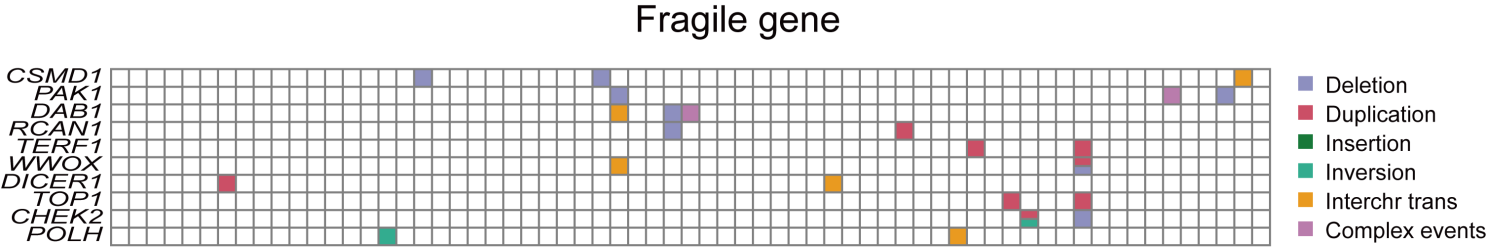


**Supplementary Figure 9. A cluster of fragile genes containing SV breakpoints in two or more UBC cases.**

**Supplementary Figure 10**


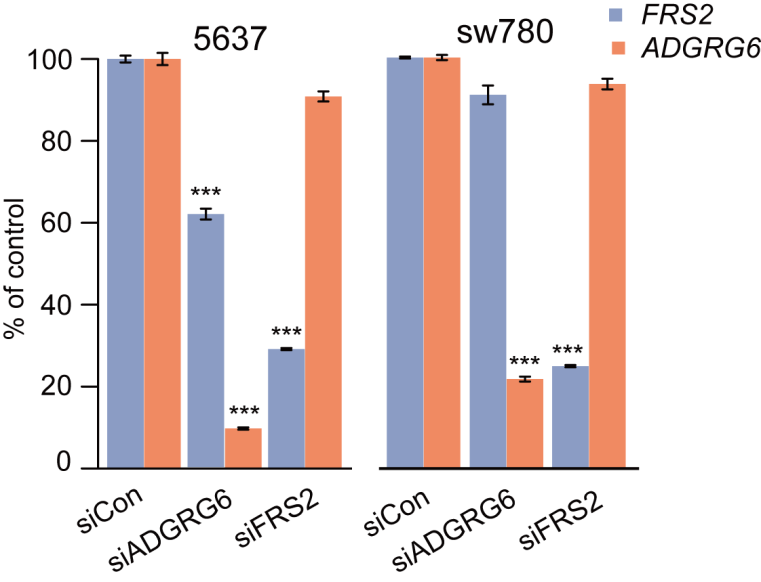


**Supplementary Figure 10. mRNA levels for *ADGRG6* and *FRS2* in the siRNA experiments.** siFRS2 has no effects on the expression of *ADGRG6* both in 5637 and SW780 cells. siADGRG6 has not effects on the expression of *FRS2* in SW780 cells, but to some extent inhibit *FRS2* expression in 5637 cells. The data shown represent averages from three independent experiments and were statistically analysed by two-sided t test. Error bars represent the SEM. * *P* < 0.05, ** *P* < 0.01, *** *P* < 0.001 versus siCon.

**Supplementary Figure 11**


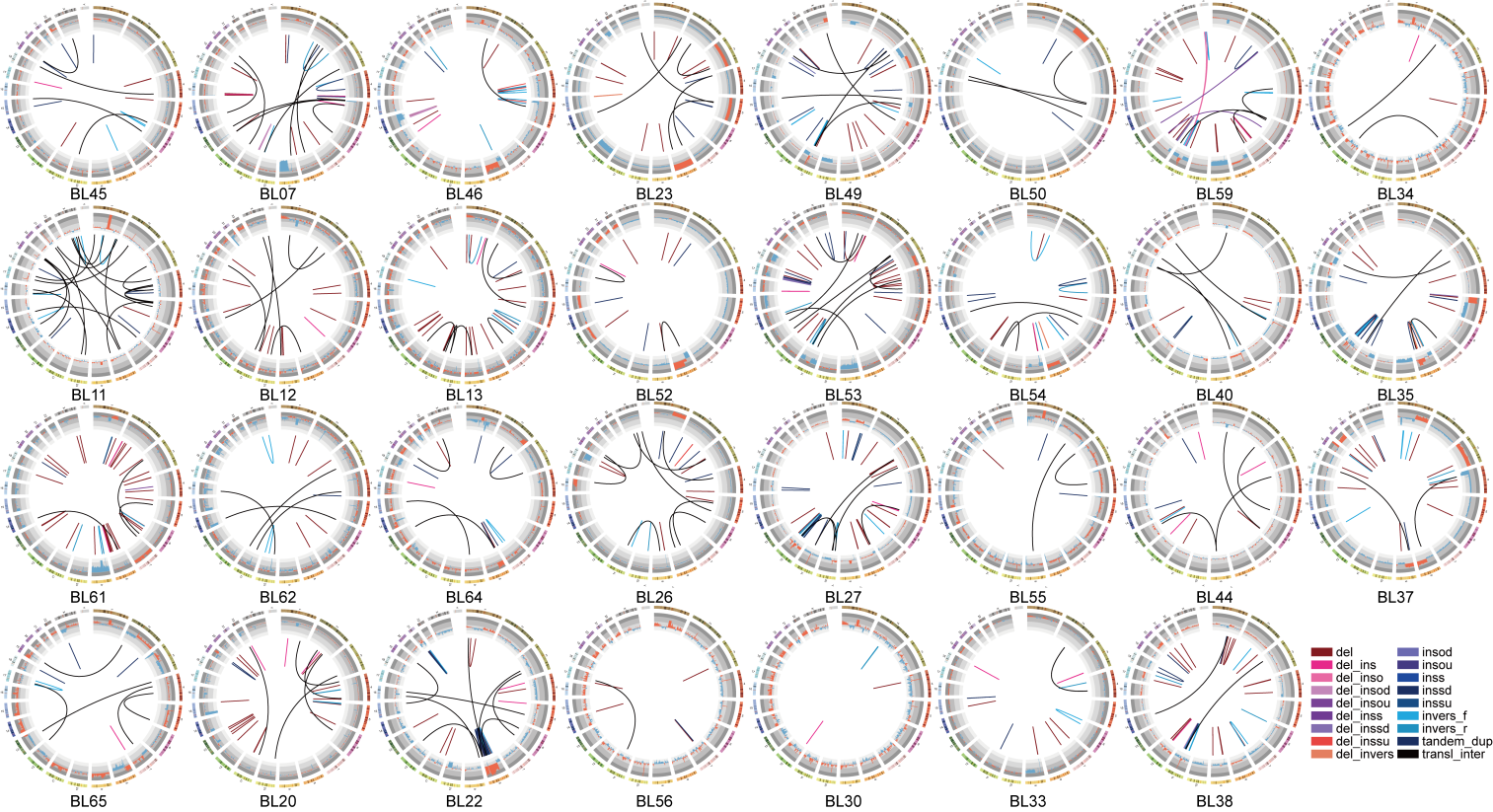


**Supplementary Figure 11. The stable subtype in UBC.** The 31 stable tumors are shown using circos. Stable tumors contain less than 50 structural rearrangements in each tumor. The chromosomes show in the outer colored rings, copy number changes are depicted in the next ring (cyan represents loss and orange represents gain). Chromosome structural rearrangements detected by whole genome paired end sequencing are represented by the inner lines, and the type of rearrangement is indicated in the legend (the abbreviations as given in the Meerkat user manual; http://compbio.med.harvard.edu/Meerkat/).

**Supplementary Figure 12**


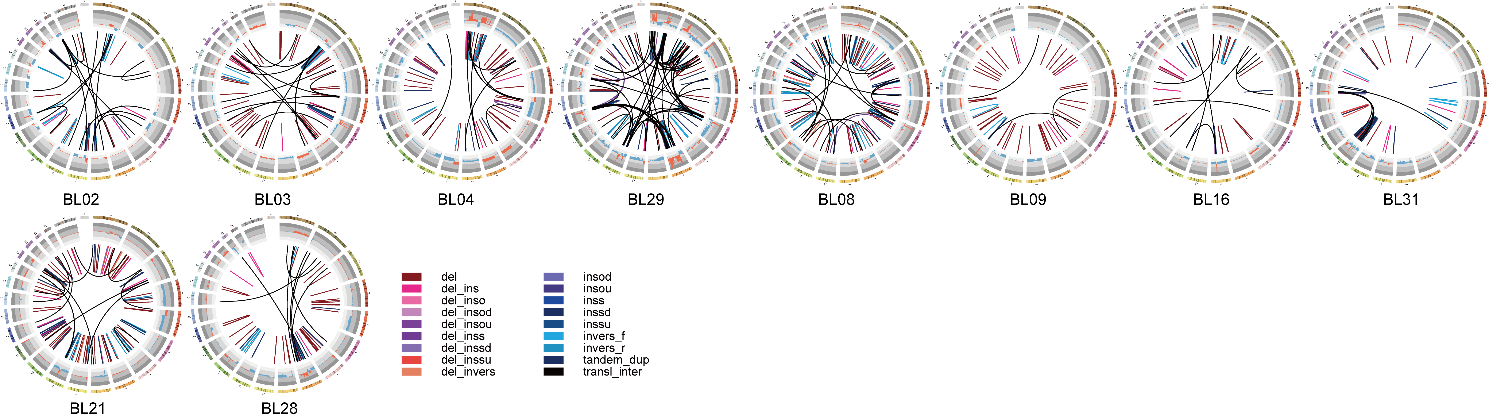


**Supplementary Figure 12. The locally rearranged subtype in UBC.** The 10 locally rearranged tumors are shown using circos. Over 25% of the structural rearrangements in the locally rearranged subtype are clustered on one of few chromosomes. The chromosomes show in the outer colored rings, copy number changes are depicted in the next ring (cyan represents loss and orange represents gain). Chromosome structural rearrangements detected by whole genome paired end sequencing are represented by the inner lines, and the type of rearrangement is indicated in the legend (the abbreviations as given in the Meerkat user manual; http://compbio.med.harvard.edu/Meerkat/).

**Supplementary Figure 13**


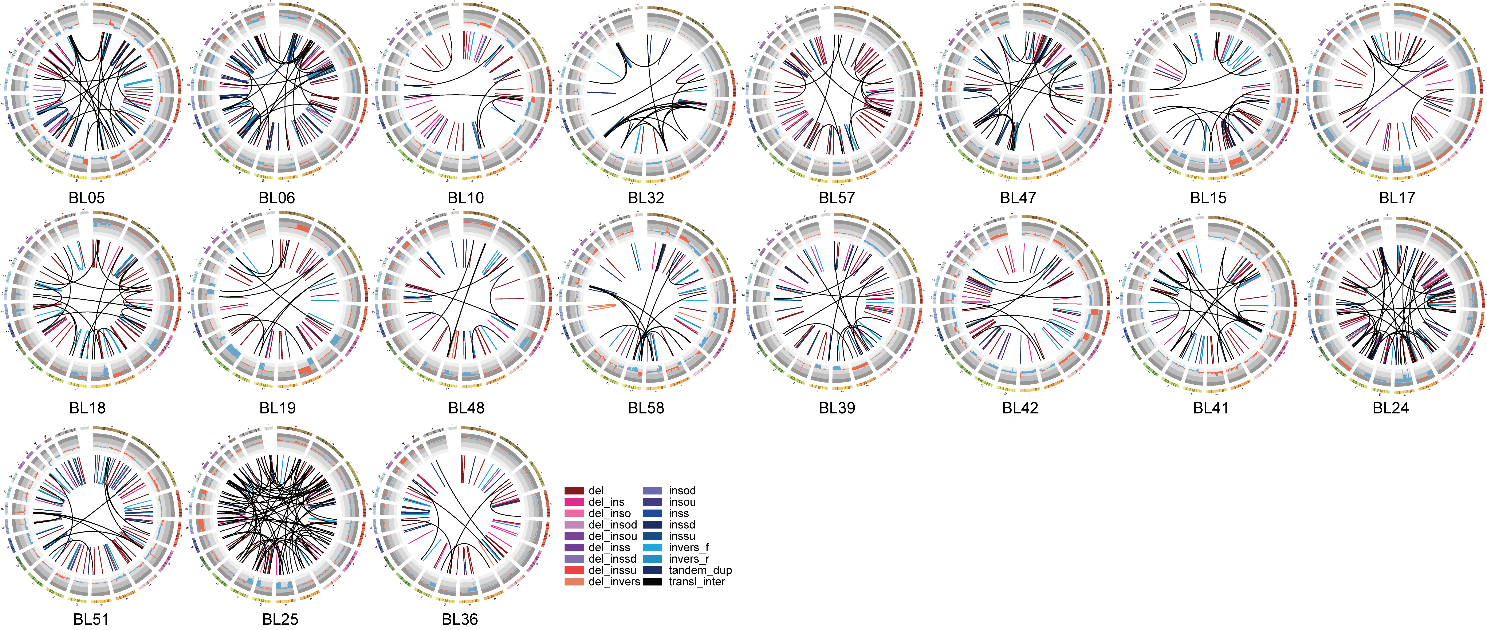


.

**Supplementary Figure 13. The scattered subtype in UBC**. The 19 tumors classified as scattered are shown using circos. The scattered tumors contained 50–200 structural rearrangements in each tumor. The chromosomes show in the outer colored rings, copy number changes are depicted in the next ring (cyan represents loss and orange represents gain). Chromosome structural rearrangements detected by whole genome paired end sequencing are represented by the inner lines, and the type of rearrangement is indicated in the legend (the abbreviations as given in the Meerkat user manual; http://compbio.med.harvard.edu/Meerkat/).

**Supplementary Figure 14**


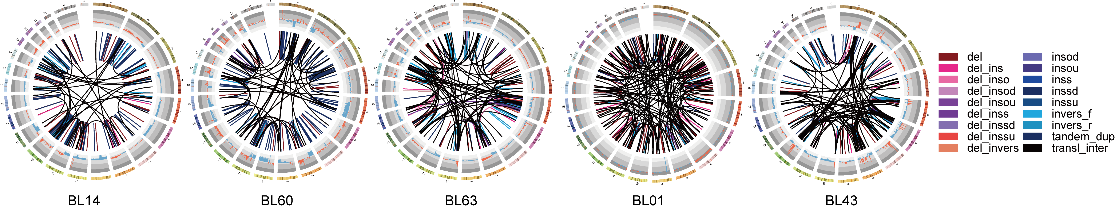


**Supplementary Figure 14. The unstable subtype in UBC.** The 5 unstable tumors are shown using circos. The unstable tumors state a large-scale of genomic instability with more than 200 SV events. The chromosomes show in the outer colored rings, copy number changes are depicted in the next ring (cyan represents loss and orange represents gain). Chromosome structural rearrangements detected by whole genome paired end sequencing are represented by the inner lines, and the type of rearrangement is indicated in the legend (the abbreviations as given in the Meerkat user manual; http://compbio.med.harvard.edu/Meerkat/).

**Supplementary Figure 15**


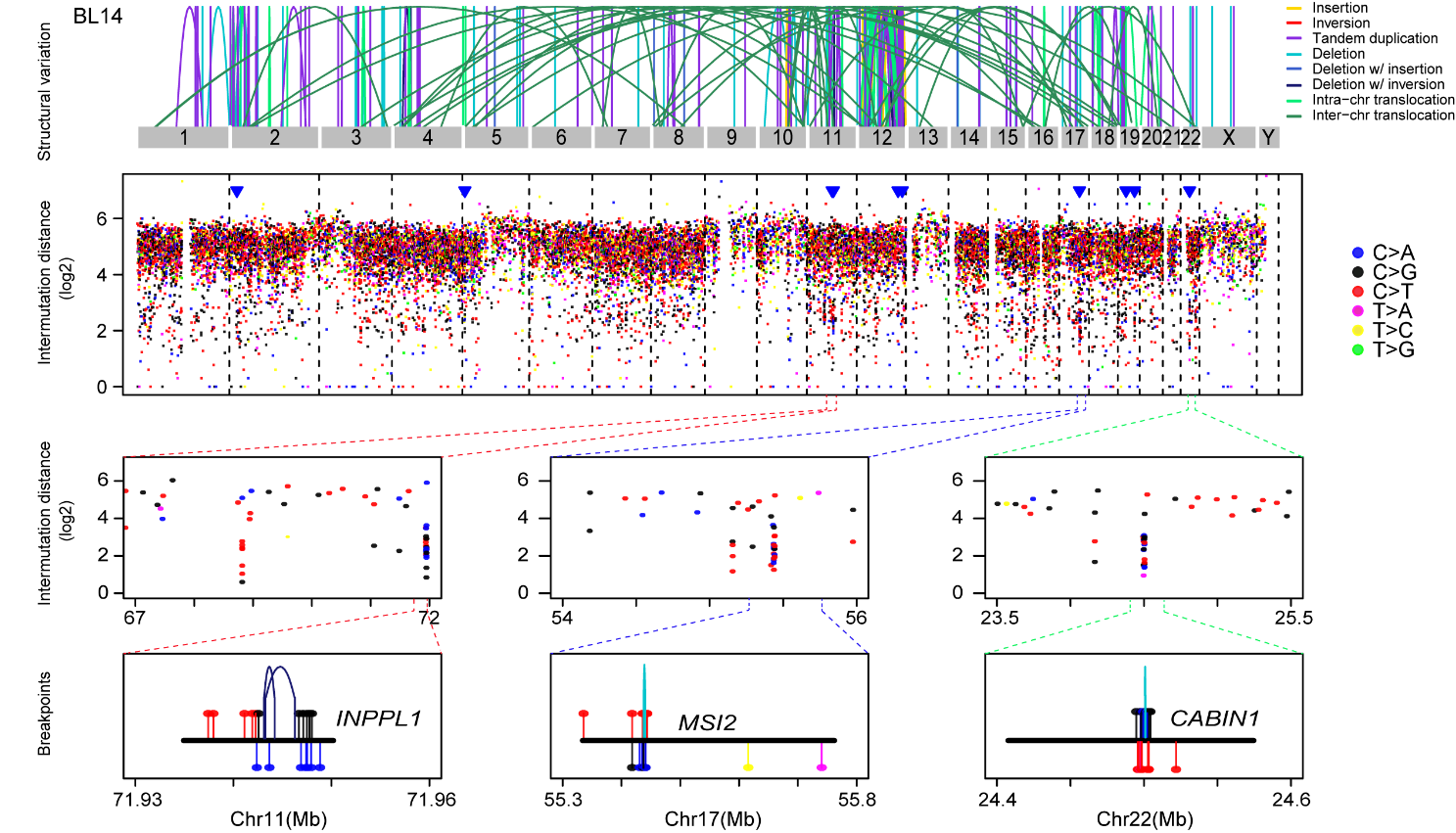


**c**

**b**

**a**

**d**

**Supplementary Figure 15. Kataegis across multi-chromosomal in UBC-BL14. a** Structural variations observed on all chromosome in BL14 are indicated by lines with different colors. The legend indicates the types of SVs. **b** Each dot of the “rainfall” plots represent a single somatic mutation ordered on the horizontal axis according to its position in genome. The vertical axis denotes the genomic distance (log2) between mutations. The upper blue triangles show the position of kataegis. **c** Highlight of kataegis region at increasing resolution to demonstrate micro-clusters within the macro-cluster in chr11:67-72M, chr17:54-56M, and chr22:23.5-25.5M. **d** Highlight of affected genes locus in each chromosome. The points and lines denotes the mutations, which are mainly C>T, C>G, C>A (the feature of signature A caused by the activation of AID/APOBEC cytidine deaminases). Three important genes (*INPPL1, MSI2* and *CABIN1*) involved in tumorigenesis are affected by kataegis.

**Supplementary Figure 16**


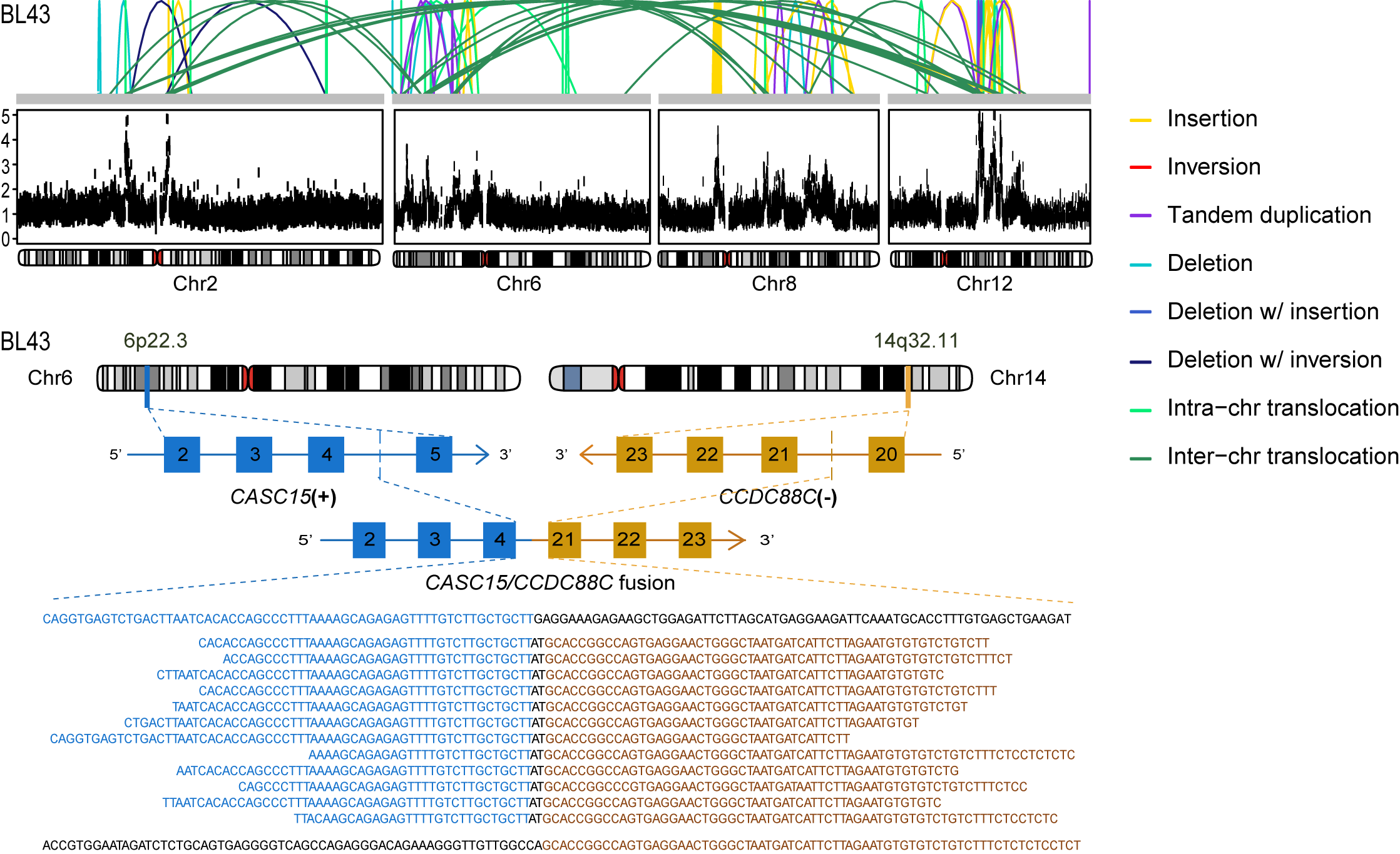


**b**

**a**

**Supplementary Figure 16. Fusion gene caused by chromothripsis-associated rearrangements in UBC. a** The representative chromothripsis map in UBC-BL43. The colored links represent the SV breakpoints in the indicated chromosomes, and the middle plots display copy number changes of the effected genomic regions. **b** Details and schematic of the CASC15/CCDC88C fusion caused by chromothripsis-associated rearrangement between chromosomes 6 and 14 in UBC-BL43. Validation of this fusion is checked by handwork. The top sequence is chr6:21856931-21857057 plus strand, and the bottom one is chr14:91797891-91798019 minus strand. The middle sequences are supported reads for the CASC15/CCDC88C fusion gene.


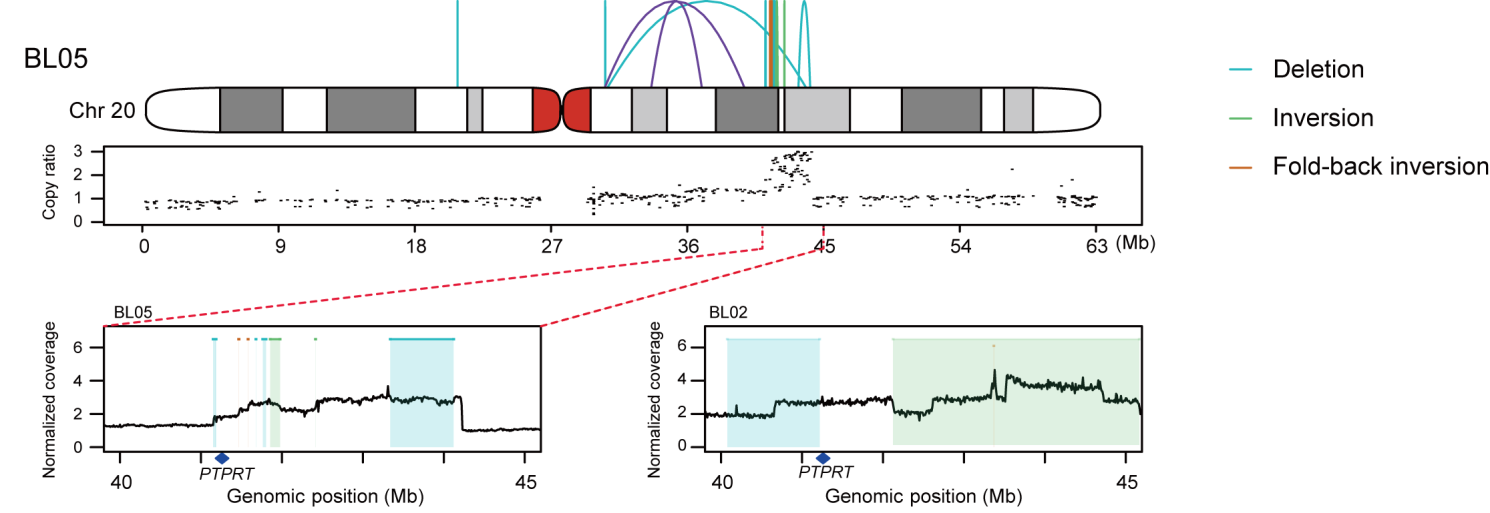
**Supplementary Figure 17**

**Supplementary Figure 17.** **BFB-associated rearrangements led to the amplification of *PTPRT* in UBC**. The upper panel details BFB event in UBC, and the bottom panel illustrates the amplification of *PTPRT* as a result of BFB in UBC-BL05 and UBC-BL02.

**Supplementary Tables**

**Supplementary Table 1. Clinical features of the 65 UBC cases**

| Sample | Sex^#^ | Age | T | N | M | TNM | NMIBC/MIBC | Grade^*^ | Primary/Relapsed |
| --- | --- | --- | --- | --- | --- | --- | --- | --- | --- |
| BL01 | F | 41 | 3 | 2 | 1 | T3N2M1 | MIBC | High | Primary |
| BL02 | M | 62 | 1 | 0 | 0 | T1N0M0 | NMIBC | Low | Primary |
| BL03 | M | 64 | 1 | 0 | 0 | T1N0M0 | NMIBC | Low | Primary |
| BL04 | M | 66 | 3 | 0 | 0 | T3N0M0 | MIBC | High | Primary |
| BL05 | M | 74 | 3 | 2 | 0 | T3N2M0 | MIBC | High | Primary |
| BL06 | M | 62 | 2 | 2 | 1 | T2N2M1 | MIBC | High | Primary |
| BL07 | M | 42 | 2 | 0 | 0 | T2N0M0 | MIBC | High | Primary |
| BL08 | M | 64 | 2 | 0 | 0 | T2N0M0 | MIBC | High | Primary |
| BL09 | F | 67 | 1 | 0 | 0 | T1N0M0 | NMIBC | High | Primary |
| BL10 | M | 71 | 2 | 0 | 0 | T2N0M0 | MIBC | High | Primary |
| BL11 | M | 74 | 3 | 1 | 0 | T3N1M0 | MIBC | High | Relapsed |
| BL12 | M | 66 | 1 | 0 | 0 | T1N0M0 | NMIBC | Low | Primary |
| BL13 | M | 40 | 4 | 0 | 0 | T4N0M0 | MIBC | High | Primary |
| BL14 | M | 56 | 2 | 0 | 0 | T2N0M0 | MIBC | High | Primary |
| BL15 | M | 75 | 2 | 0 | 0 | T2N0M0 | MIBC | High | Primary |
| BL16 | M | 61 | 2 | 0 | 0 | T2N0M0 | MIBC | High | Primary |
| BL17 | M | 53 | 4 | 0 | 0 | T4N0M0 | MIBC | High | Primary |
| BL18 | M | 65 | 4 | 0 | 0 | T4N0M0 | MIBC | High | Relapsed |
| BL19 | M | 66 | 3 | 0 | 0 | T3N0M0 | MIBC | High | Primary |
| BL20 | M | 43 | 1 | 0 | 0 | T1N0M0 | NMIBC | Low | Primary |
| BL21 | M | 54 | 2 | 0 | 0 | T2N0M0 | MIBC | High | Primary |
| BL22 | M | 85 | 2 | 0 | 0 | T2N0M0 | MIBC | High | Primary |
| BL23 | M | 65 | 2 | 0 | 0 | T2N0M0 | MIBC | High | Primary |
| BL24 | M | 62 | 2 | 0 | 0 | T2N0M0 | MIBC | High | Primary |
| BL25 | M | 68 | 1 | 0 | 0 | T1N0M0 | NMIBC | Low | Primary |
| BL26 | F | 56 | 1 | 0 | 0 | T1N0M0 | NMIBC | Low | Primary |
| BL27 | M | 59 | 2 | 0 | 0 | T2N0M0 | MIBC | High | Primary |
| BL28 | M | 70 | 3 | 0 | 0 | T3N0M0 | MIBC | High | Primary |
| BL29 | M | 57 | 1 | 0 | 0 | T1N0M0 | NMIBC | Low | Relapsed |
| BL30 | F | 89 | 2 | 0 | 0 | T2N0M0 | MIBC | High | Primary |
| BL31 | F | 63 | 1 | 0 | 0 | T1N0M0 | NMIBC | Low | Primary |
| BL32 | M | 62 | 3 | 0 | 0 | T3N0M0 | MIBC | High | Primary |
| BL33 | M | 70 | 1 | 0 | 0 | T1N0M0 | NMIBC | Low | Primary |
| BL34 | M | 65 | 2 | 0 | 0 | T2N0M0 | MIBC | High | Primary |
| BL35 | M | 50 | 1 | 0 | 0 | T1N0M0 | NMIBC | Low | Primary |
| BL36 | M | 64 | 1 | 0 | 0 | T1N0M0 | NMIBC | Low | Primary |
| BL37 | F | 62 | 3 | 0 | 1 | T3N0M1 | MIBC | High | Primary |
| BL38 | M | 58 | 2 | 0 | 0 | T2N0M0 | MIBC | High | Primary |
| BL39 | M | 59 | 1 | 0 | 0 | T1N0M0 | NMIBC | Low | Primary |
| BL40 | M | 75 | 2 | 0 | 0 | T2N0M0 | MIBC | High | Primary |
| BL41 | M | 78 | 2 | 0 | 0 | T2N0M0 | MIBC | High | Primary |
| BL42 | M | 58 | 2 | 0 | 0 | T2N0M0 | MIBC | High | Primary |
| BL43 | M | 76 | 2 | 0 | 0 | T2N0M0 | MIBC | High | Primary |
| BL44 | M | 52 | 3 | 0 | 0 | T3N0M0 | MIBC | High | Primary |
| BL45 | M | 70 | 1 | 0 | 0 | T1N0M0 | NMIBC | Low | Primary |
| BL46 | M | 69 | 1 | 0 | 0 | T1N0M0 | NMIBC | Low | Primary |
| BL47 | M | 72 | a | 0 | 0 | TaN0M0 | NMIBC | Low | Primary |
| BL48 | M | 80 | a | 0 | 0 | TaN0M0 | NMIBC | PUNLMP | Primary |
| BL49 | M | 78 | a | 0 | 0 | TaN0M0 | NMIBC | PUNLMP | Primary |
| BL50 | M | 54 | a | 0 | 0 | TaN0M0 | NMIBC | PUNLMP | Primary |
| BL51 | M | 64 | a | 0 | 0 | TaN0M0 | NMIBC | PUNLMP | Primary |
| BL52 | M | 65 | a | 0 | 0 | TaN0M0 | NMIBC | PUNLMP | Primary |
| BL53 | M | 84 | a | 0 | 0 | TaN0M0 | NMIBC | Low | Primary |
| BL54 | M | 57 | a | 0 | 0 | TaN0M0 | NMIBC | PUNLMP | Primary |
| BL55 | M | 24 | 1 | 0 | 0 | T1N0M0 | NMIBC | Low | Primary |
| BL56 | M | 65 | 1 | 0 | 0 | T1N0M0 | NMIBC | Low | Primary |
| BL57 | M | 65 | 1 | 0 | 0 | T1N0M0 | NMIBC | Low | Primary |
| BL58 | M | 59 | 1 | 0 | 0 | T1N0M0 | NMIBC | Low | Primary |
| BL59 | M | 48 | 1 | 0 | 0 | T1N0M0 | NMIBC | Low | Primary |
| BL60 | M | 63 | 2 | 0 | 0 | T2N0M0 | MIBC | High | Primary |
| BL61 | M | 63 | 1 | 0 | 0 | T1N0M0 | NMIBC | Low | Primary |
| BL62 | M | 73 | 2 | 0 | 0 | T2N0M0 | MIBC | High | Relapsed |
| BL63 | F | 61 | 1 | 0 | 0 | T1N0M0 | NMIBC | High | Primary |
| BL64 | M | 40 | 1 | 0 | 0 | T1N0M0 | NMIBC | Low | Primary |
| BL65 | M | 66 | 1 | 0 | 0 | T1N0M0 | NMIBC | Low | Primary |

^#^ M, Male; F, Female.

^*^PUNLMP, papillary urothelial malignancy of low malignant potential; Low, low-grade papillary urothelial carcinoma; High, high-grade papillary urothelial carcinoma (WHO/ISUP 2004 classification).

**Supplementary Table 2. Correlations between the number of mutations and clinical phenotypes**

|  | *P value** | | | | | |  | Correlation coefficient* | | | | | |
| --- | --- | --- | --- | --- | --- | --- | --- | --- | --- | --- | --- | --- | --- |
|  | Point mutation | SV | Indel | Indel in exonic | SNV | SNV in exonic |  | Point mutation | SV | Indel | Indel in exonic | SNV | SNV in exonic |
| sex | 0.8648 | 0.4504 | 0.976 | 0.9131 | 0.6958 | 0.9887 |  | 0.0215 | -0.0953 | -0.0038 | 0.0138 | 0.0494 | 0.0018 |
| Age | 0.7546 | 0.4735 | 0.7198 | 0.9006 | 0.9223 | 0.6452 |  | -0.0395 | -0.0905 | -0.0454 | -0.0158 | -0.0123 | -0.0582 |
| T | 0.7868 | 0.3621 | 0.5878 | 0.8533 | 0.6099 | 0.738 |  | -0.0342 | 0.1149 | 0.0685 | -0.0234 | -0.0645 | -0.0423 |
| N | 0.7924 | 0.2905 | 0.783 | 0.7804 | 0.6077 | 0.7452 |  | -0.0333 | 0.1331 | 0.0348 | -0.0353 | -0.0649 | -0.0411 |
| M | 0.7726 | 0.3642 | 0.8857 | 0.8492 | 0.5889 | 0.7262 |  | -0.0365 | 0.1144 | 0.0182 | -0.0241 | -0.0683 | -0.0443 |
| NMIBC/MIBC | 0.8215 | 0.0954 | 0.5541 | 0.6758 | 0.7043 | 0.8848 |  | 0.0285 | -0.2086 | -0.0747 | -0.0529 | 0.048 | 0.0183 |
| Primary/Relapsed | 0.9205 | 0.9229 | 0.7458 | 0.4168 | 0.9323 | 0.9807 |  | -0.0126 | 0.0122 | 0.041 | 0.1024 | 0.0108 | 0.0031 |

* *P* values and correlation coefficients were calculated using Pearson's correlation test and Bonferroni's correction.

**Supplementary Table 3. Similarities between the signatures in this study and COSMIC databases**

| UBC WGS | COSMIC* | Cosine similarity | Note* |
| --- | --- | --- | --- |
| Signature A | Signature 2 | 0.8446 | Mutations of similar patterns to Signatures 2 are commonly observed in the phenomenon of local hypermutation present in some cancers, known as kataegis, and activation of AID/APOBEC cytidine deaminases might be potentially implicated in this process. |
| Signature B | Signature 22 | 0.9092 | Signature 22 has been found in cancer samples with known exposures to aristolochic acid. |
| Signature C | Signature 5 | 0.9136 | Signature 5 has been found in most cancer types and its aetiology is unknown. |
| Signature D | Signature 18 | 0.6554 | Signature 18 has been found commonly in neuroblastoma. In addition, signature 18 has been also found in stomach and breast carcinomas, and its aetiology remains unknown. |
|  | Signature 8 | 0.6531 | Signature 8 has been found in breast cancer and medulloblastoma. Its aetiology remains unknown. |
|  | Signature 4 | 0.6436 | Signature 4 is associated with smoking and its profile is similar to the mutational pattern found in experimental systems exposed to tobacco carcinogens. |
|  | Signature 29 | 0.6038 | Signature 29 has been found in tobacco-related cancers. |

* Reference: Alexandrov, L. B. et al. Signatures of mutational processes in human cancer. Nature 500, 415-421, doi:10.1038/nature12477 (2013).

**Supplementary Table 4. Associations of mutational signatures with genetic alterations and clinical background**

|  | Clinical parameter or mutated genes | Simple linear regression analysis | Multiple linear regression analysis |
| --- | --- | --- | --- |
| Signature A | BFB | 0.0111* | 0.8102 |
|  | *CASP8* | 0.0405* | 0.0063* |
|  | *ADGRG6* | 0.0208* | 0.0003* |
|  | *PDE4D* | 0.0127* | 0.5049 |
|  | *PLEKHS1* | 0.0159* | 0.8459 |
|  | *PLXDC1* | 0.0001*** | 0.6223 |
|  | *RB1* | 0.0003*** | 0.3975 |
|  | *TBC1D12* | 0.0020** | 0.9604 |
|  | *VPS13B* | 0.0158* | 0.7945 |
|  | *WDR74* | 0.0224* | 0.6831 |
| Signature B | Age | 0.4595 | 0.7191 |
|  | *DDR2* | 0.0490* | 0.1546 |
|  | *ERCC4* | 0.0168* | 0.0642 |
|  | *FHIT* | 0.0466* | 0.4483 |
|  | *GPC5* | 0.0079** | 0.1046 |
|  | *ADGRG6* | 0.0042** | 0.0140* |
|  | M | 0.3424 | 0.0687 |
|  | N | 0.3957 | 0.0682 |
|  | Sex | 0.1946 | 0.6672 |
|  | T | 0.3787 | 0.6597 |
|  | *TP53* | 0.0474* | 0.1696 |
| Signature C | BFB | 0.0037** | 0.1809 |
|  | M | 0.3968 | 0.1342 |
|  | N | 0.4271 | 0.1078 |
|  | P/R | 0.4893 | 0.6837 |
|  | *PDE4D* | 0.0053** | 0.1925 |
|  | *PLXDC1* | 0.0022** | 0.7645 |
|  | *RB1* | 0.0029** | 0.6933 |
|  | *TBC1D12* | 0.0083** | 0.2859 |
|  | *TERT* | 0.0276* | 0.359 |
|  | *VPS13B* | 0.0152* | 0.8218 |
|  | *WDR74* | 0.0198* | 0.4352 |
| Signature D | Age | 0.3162 | 0.575 |
|  | BFB | 0.0199* | 0.2694 |
|  | *ADGRG6* | 0.0496* | 0.0919 |
|  | M | 0.0782 | 0.359 |
|  | N | 0.0583 | 0.0323* |
|  | P/R | 0.2182 | 0.8847 |
|  | *PDE4D* | 0.0370* | 0.4884 |
|  | *PLXDC1* | 0.0311* | 0.5813 |
|  | *RB1* | 0.0370* | 0.8367 |
|  | Sex | 0.4011 | 0.5364 |
|  | T | 0.2416 | 0.0634 |
|  | *TBC1D12* | 0.0219* | 0.4323 |
|  | *VPS13B* | 0.0425* | 0.5416 |

Significant codes: * *P* < 0.05, ** *P* < 0.01, *** *P* < 0.001.

**Supplementary Table 5. Significant mutated non-coding regulatory regions in the 65 UBC cases**

| Chrom | Region_start | Region_end | Gene_symbol | SNV number | Functional | *B-H adjusted *P* value compared to background | *B-H adjusted *P* value compared to whole genome | Recurrence of patients |
| --- | --- | --- | --- | --- | --- | --- | --- | --- |
| chr5 | 1,295,104 | 1,295,684 | *TERT* | 6 | Promoter | 1.70E-04 | 8.89E-05 | 5 |
| chr6 | 142,705,654 | 142,706,390 | *ADGRG6* | 17 | Enhancer | 1.23E-26 | 4.74E-24 | 15 |
| chr7 | 66,204,712 | 66,205,919 | *KCTD7* | 6 | Promoter | 1.97E-03 | 3.23E-03 | 5 |
| chr7 | 66,205,519 | 66,206,472 | *RABGEF1* | 5 | Promoter | 1.22E-02 | 1.58E-02 | 4 |
| chr8 | 29,952,153 | 29,953,028 | *LEPROTL1* | 6 | Promoter | 2.27E-04 | 6.62E-04 | 5 |
| chr8 | 29,952,913 | 29,953,028 | *LEPROTL1* | 6 | UTR | 2.96E-09 | 1.10E-08 | 5 |
| chr10 | 115,511,012 | 115,511,640 | *PLEKHS1* | 9 | Promoter | 1.15E-11 | 1.13E-09 | 9 |
| chr10 | 96,161,837 | 96,162,370 | *TBC1D12* | 9 | Promoter | 5.34E-12 | 5.25E-10 | 8 |
| chr10 | 96,162,260 | 96,162,370 | *TBC1D12* | 9 | UTR | 7.32E-18 | 7.41E-16 | 8 |
| chr11 | 44,722,205 | 44,722,452 | *TSPAN18* | 4 | Enhancer | 9.35E-03 | 6.81E-03 | 4 |
| chr11 | 62,607,042 | 62,610,390 | *WDR74* | 12 | Promoter | 1.23E-07 | 3.58E-07 | 8 |
| chr11 | 62,609,040 | 62,609,281 | *WDR74* | 8 | UTR | 2.40E-11 | 5.00E-11 | 6 |
| chr11 | 62,609,040 | 62,609,281 | *WDR74* | 8 | UTR | 2.40E-11 | 5.00E-11 | 6 |
| chr17 | 37,308,933 | 37,311,106 | *PLXDC1* | 8 | Promoter | 5.13E-04 | 4.98E-04 | 4 |
| chr19 | 1,382,946 | 1,383,925 | *NDUFS7* | 5 | Promoter | 1.93E-02 | 1.68E-02 | 4 |

*B-H adjusted *P* value: Benjamini-Hochberg adjusted *P* value (binomial test).

**Supplementary Table 6. Clinical features of the additional 196 UBC cases**

| Sample | Sex^#^ | Age | TNM | | | NMIBC/ MIBC | Grade^*^ | Primary/ Relapsed | Surgical approach | *FRS2* copy number | | *ADGRG6* enhancer mutation | | Overall survival (month) | Survival status^&^ |
| --- | --- | --- | --- | --- | --- | --- | --- | --- | --- | --- | --- | --- | --- | --- | --- |
|  |  |  | T | N | M |  |  |  |  |  |  | G>A  (g. 142,706,206) | C>T/G  (g. 142,706,209) |  |  |
| BC_T001 | M | 68 | 3 | 2 | 0 | MIBC | High | Relapsed | Radical cystectomy | 1 | 1.095 |  |  | 19 | 0 |
| BC_T002 | M | 63 | 1 | 0 | 0 | NMIBC | Low | Relapsed | Transurethral resection | 2 | 2.075 |  |  | 36 | 0 |
| BC_T003 | M | 59 | 3 | 1 | 0 | MIBC | Low | Primary | Radical cystectomy | 1 | 1.206 | G>A |  | 18 | 0 |
| BC_T004 | M | 48 | a | 0 | 0 | NMIBC | Low | Primary | Transurethral resection | 2 | 2.401 | G>A |  | 48 | 0 |
| BC_T005 | F | 57 | a | 0 | 0 | NMIBC | Low | Primary | Transurethral resection | 4 | 3.968 |  |  | 41 | 0 |
| BC_T006 | F | 60 | a | 0 | 0 | NMIBC | Low | Primary | Transurethral resection | 3 | 2.962 | G>A |  | NA | NA |
| BC_T007 | M | 74 | a | 0 | 0 | NMIBC | PUNLMP | Primary | Transurethral resection | 2 | 2.391 | G>A |  | 37 | 1 |
| BC_T008 | M | 51 | 3 | 1 | 0 | MIBC | High | Primary | Radical cystectomy | 2 | 2.109 |  |  | 12 | 1 |
| BC_T009 | M | 64 | 1 | 0 | 0 | NMIBC | High | Primary | Transurethral resection | 3 | 2.869 |  |  | 47 | 0 |
| BC_T010 | M | 70 | a | 0 | 0 | NMIBC | Low | Primary | Transurethral resection | 2 | 1.897 | G>A |  | NA | NA |
| BC_T011 | M | 79 | 1 | 0 | 0 | NMIBC | High | Primary | Transurethral resection | 5 | 4.975 |  | C>G | 18 | 1 |
| BC_T012 | M | 80 | a | 0 | 0 | NMIBC | PUNLMP | Primary | Transurethral resection | 2 | 1.568 |  |  | 45 | 0 |
| BC_T013 | M | 73 | a | 0 | 0 | NMIBC | PUNLMP | Primary | Transurethral resection | 2 | 2.275 |  |  | 57 | 0 |
| BC_T014 | M | 78 | a | 0 | 0 | NMIBC | Low | Primary | Transurethral resection | 2 | 1.968 |  |  | 55 | 0 |
| BC_T015 | M | 67 | a | 0 | 0 | NMIBC | PUNLMP | Primary | Transurethral resection | 4 | 4.051 |  |  | 42 | 1 |
| BC_T016 | M | 74 | a | 0 | 0 | NMIBC | High | Primary | Transurethral resection | 2 | 1.948 |  | C>G | 19 | 1 |
| BC_T017 | F | 70 | a | 0 | 0 | NMIBC | PUNLMP | Primary | Transurethral resection | 2 | 2.343 |  |  | 56 | 0 |
| BC_T018 | M | 50 | 2 | 0 | 0 | MIBC | High | Primary | Radical cystectomy | 2 | 1.911 |  | C>G | 38 | 0 |
| BC_T019 | M | 80 | a | 0 | 0 | NMIBC | PUNLMP | Primary | Transurethral resection | 2 | 2.417 |  | C>T | 47 | 0 |
| BC_T020 | M | 81 | a | 0 | 0 | NMIBC | High | Primary | Transurethral resection | 2 | 2.317 |  | C>T | 32 | 1 |
| BC_T021 | M | 65 | a | 0 | 0 | NMIBC | Low | Primary | Transurethral resection | 2 | 1.911 | G>A |  | 43 | 0 |
| BC_T022 | M | 102 | 2 | 0 | 0 | MIBC | High | Primary | Transurethral resection | 2 | 1.941 | G>A | C>G | 11 | 1 |
| BC_T023 | M | 73 | 2 | 0 | 0 | MIBC | High | Primary | Radical cystectomy | 2 | 2.442 |  | C>G | 47 | 0 |
| BC_T024 | M | 74 | 1 | 0 | 0 | NMIBC | Low | Primary | Transurethral resection | 2 | 1.977 |  |  | NA | NA |
| BC_T025 | M | 50 | 2 | 0 | 0 | MIBC | High | Primary | Radical cystectomy | 2 | 1.854 |  |  | 25 | 1 |
| BC_T026 | M | 84 | 1 | 0 | 0 | NMIBC | Low | Relapsed | Transurethral resection | 1 | 1.240 | G>A |  | 12 | 0 |
| BC_T027 | M | 69 | 1 | 0 | 0 | NMIBC | High | Primary | Transurethral resection | 1 | 1.364 |  |  | 18 | 0 |
| BC_T028 | F | 73 | 1 | 0 | 0 | NMIBC | PUNLMP | Primary | Transurethral resection | 1 | 1.278 | G>A |  | 30 | 0 |
| BC_T029 | M | 40 | 1 | 0 | 0 | NMIBC | High | Primary | Transurethral resection | 2 | 1.506 |  | C>T | NA | NA |
| BC_T030 | M | 62 | 1 | 0 | 0 | NMIBC | Low | Primary | Transurethral resection | 1 | 1.132 | G>A |  | 30 | 0 |
| BC_T031 | M | 81 | a | 0 | 0 | NMIBC | Low | Primary | Transurethral resection | 3 | 3.276 |  | C>T | 30 | 0 |
| BC_T032 | M | 60 | 1 | 0 | 0 | NMIBC | High | Primary | Transurethral resection | 2 | 2.237 | G>A |  | 27 | 0 |
| BC_T033 | M | 57 | 1 | 0 | 0 | NMIBC | Low | Primary | Transurethral resection | 2 | 2.035 |  |  | 16 | 0 |
| BC_T034 | F | 70 | 1 | 0 | 0 | NMIBC | Low | Relapsed | Transurethral resection | 1 | 1.378 |  |  | 30 | 0 |
| BC_T035 | M | 88 | 1 | 0 | 0 | NMIBC | High | Primary | Transurethral resection | 2 | 2.142 | G>A | C>G | 13 | 1 |
| BC_T036 | M | 68 | 2 | 0 | 0 | MIBC | High | Primary | Radical cystectomy | 2 | 2.125 | G>A | C>T | 16 | 0 |
| BC_T037 | F | 59 | 4 | 0 | 0 | MIBC | Low | Primary | Radical cystectomy | 1 | 1.339 |  |  | 14 | 0 |
| BC_T038 | M | 71 | 3 | 0 | 0 | MIBC | High | Relapsed | Radical cystectomy | 2 | 1.651 |  |  | 23 | 0 |
| BC_T039 | M | 82 | 2 | 0 | 0 | MIBC | High | Relapsed | Transurethral resection | 3 | 3.058 |  |  | 24 | 1 |
| BC_T040 | F | 83 | 1 | 0 | 0 | NMIBC | High | Primary | Radical cystectomy | 9 | 8.552 | G>A |  | 12 | 1 |
| BC_T041 | M | 78 | 4 | 0 | 0 | MIBC | High | Primary | Radical cystectomy | 1 | 1.143 |  |  | 12 | 0 |
| BC_T042 | M | 74 | 3 | 0 | 0 | MIBC | High | Primary | Radical cystectomy | 2 | 1.633 |  |  | 20 | 0 |
| BC_T043 | M | 52 | 1 | 0 | 0 | NMIBC | High | Primary | Radical cystectomy | 2 | 1.726 |  |  | 19 | 0 |
| BC_T044 | F | 53 | 1 | 0 | 0 | NMIBC | Low | Relapsed | Transurethral resection | 1 | 0.983 |  |  | 12 | 0 |
| BC_T045 | F | 81 | 1 | 0 | 0 | NMIBC | High | Relapsed | Radical cystectomy | 1 | 1.114 |  |  | 12 | 0 |
| BC_T046 | M | 73 | 1 | 0 | 0 | NMIBC | High | Relapsed | Radical cystectomy | 1 | 1.248 |  | C>G | 16 | 0 |
| BC_T047 | F | 68 | 1 | 0 | 0 | NMIBC | High | Primary | Transurethral resection | 2 | 1.586 |  |  | 48 | 0 |
| BC_T048 | M | 66 | a | 0 | 0 | NMIBC | PUNLMP | Primary | Transurethral resection | 2 | 2.367 |  | C>T | 34 | 0 |
| BC_T049 | M | 61 | a | 0 | 0 | NMIBC | Low | Primary | Transurethral resection | 2 | 1.689 |  |  | 47 | 0 |
| BC_T050 | F | 76 | 2 | 0 | 0 | MIBC | High | Primary | Transurethral resection | 1 | 1.043 |  | C>T | 30 | 1 |
| BC_T051 | M | 73 | 1 | 0 | 0 | NMIBC | High | Primary | Transurethral resection | 2 | 2.451 |  | C>T | NA | NA |
| BC_T052 | M | 64 | 2 | 0 | 0 | MIBC | High | Primary | Radical cystectomy | 1 | 1.102 | G>A |  | 31 | 1 |
| BC_T053 | M | 75 | 1 | 0 | 0 | NMIBC | Low | Primary | Transurethral resection | 2 | 1.551 | G>A | C>T | 39 | 1 |
| BC_T054 | M | 62 | a | 0 | 0 | NMIBC | PUNLMP | Primary | Transurethral resection | 2 | 1.877 |  |  | 59 | 0 |
| BC_T055 | F | 65 | 2 | 0 | 0 | MIBC | Low | Primary | Transurethral resection | 2 | 1.740 |  |  | 48 | 0 |
| BC_T056 | M | 74 | a | 0 | 0 | NMIBC | PUNLMP | Primary | Transurethral resection | 2 | 1.949 |  | C>T | 39 | 0 |
| BC_T057 | M | 80 | 1 | 0 | 0 | NMIBC | High | Primary | Transurethral resection | 2 | 1.818 | G>A |  | 33 | 1 |
| BC_T058 | F | 63 | a | 0 | 0 | NMIBC | Low | Primary | Transurethral resection | 2 | 2.245 |  | C>T | 42 | 0 |
| BC_T059 | F | 59 | a | 0 | 0 | NMIBC | High | Primary | Transurethral resection | 3 | 3.322 |  |  | 41 | 0 |
| BC_T060 | F | 70 | a | 0 | 0 | NMIBC | PUNLMP | Primary | Transurethral resection | 3 | 2.535 | G>A | C>T | 29 | 0 |
| BC_T061 | M | 30 | a | 0 | 0 | NMIBC | PUNLMP | Primary | Transurethral resection | 2 | 1.645 | G>A |  | 58 | 1 |
| BC_T062 | M | 67 | 2 | 0 | 0 | MIBC | High | Primary | Transurethral resection | 24 | 24.230 |  |  | 18 | 1 |
| BC_T063 | F | 67 | a | 0 | 0 | NMIBC | High | Primary | Transurethral resection | 4 | 3.885 | G>A | C>T | 19 | 1 |
| BC_T064 | F | 78 | 2 | 0 | 0 | MIBC | High | Primary | Radical cystectomy | 2 | 1.777 | G>A |  | 34 | 1 |
| BC_T065 | M | 76 | a | 0 | 0 | NMIBC | High | Primary | Transurethral resection | 2 | 2.136 | G>A |  | 39 | 1 |
| BC_T066 | M | 78 | a | 0 | 0 | NMIBC | High | Primary | Transurethral resection | 3 | 2.936 | G>A |  | 31 | 0 |
| BC_T067 | M | 49 | a | 0 | 0 | NMIBC | PUNLMP | Primary | Transurethral resection | 5 | 4.645 | G>A | C>T | 38 | 1 |
| BC_T068 | M | 83 | 1 | 0 | 0 | NMIBC | High | Primary | Transurethral resection | 3 | 3.336 | G>A |  | 17 | 1 |
| BC_T069 | M | 61 | a | 0 | 0 | NMIBC | Low | Primary | Transurethral resection | 3 | 2.735 | G>A |  | 41 | 0 |
| BC_T070 | F | 59 | a | 0 | 0 | NMIBC | Low | Primary | Transurethral resection | 3 | 3.077 | G>A | C>G | 41 | 0 |
| BC_T071 | M | 69 | 2 | 0 | 0 | MIBC | High | Primary | Radical cystectomy | 3 | 2.741 |  |  | 60 | 0 |
| BC_T072 | M | 54 | a | 0 | 0 | NMIBC | Low | Primary | Transurethral resection | 5 | 5.411 |  | C>T | 37 | 0 |
| BC_T073 | M | 77 | 1 | 0 | 0 | NMIBC | Low | Primary | Transurethral resection | 4 | 3.652 |  |  | 16 | 1 |
| BC_T074 | M | 59 | a | 0 | 0 | NMIBC | PUNLMP | Primary | Transurethral resection | 3 | 2.963 | G>A |  | 37 | 0 |
| BC_T075 | M | 58 | 2 | 0 | 0 | MIBC | High | Primary | Radical cystectomy | 1 | 1.456 | G>A |  | 14 | 1 |
| BC_T076 | M | 61 | 4 | 1 | X | MIBC | High | Relapsed | Radical cystectomy | 2 | 1.794 | G>A |  | 5 | 1 |
| BC_T077 | M | 53 | 1 | 0 | 0 | NMIBC | High | Primary | Radical cystectomy | 1 | 1.421 |  |  | 14 | 0 |
| BC_T078 | M | 74 | 3 | 0 | X | MIBC | High | Primary | Radical cystectomy | 2 | 1.593 |  |  | 16 | 0 |
| BC_T079 | M | 74 | 1 | 0 | 0 | NMIBC | High | Primary | Transurethral resection | 2 | 1.816 | G>A |  | 34 | 1 |
| BC_T080 | F | 52 | 2 | 0 | 0 | MIBC | High | Primary | Radical cystectomy | 2 | 2.069 |  |  | 72 | 0 |
| BC_T081 | M | 68 | a | 0 | 0 | NMIBC | PUNLMP | Primary | Transurethral resection | 2 | 1.822 |  |  | 62 | 0 |
| BC_T082 | M | 79 | a | 0 | 0 | NMIBC | PUNLMP | Primary | Transurethral resection | 2 | 1.835 |  |  | 56 | 1 |
| BC_T083 | M | 58 | 1 | 0 | 0 | NMIBC | High | Primary | Transurethral resection | 1 | 1.447 |  |  | 51 | 0 |
| BC_T084 | M | 76 | a | 0 | 0 | NMIBC | PUNLMP | Primary | Transurethral resection | 2 | 1.806 |  |  | 78 | 0 |
| BC_T085 | F | 53 | a | 0 | 0 | NMIBC | High | Primary | Transurethral resection | 1 | 1.483 |  |  | 77 | 0 |
| BC_T086 | M | 55 | 1 | 0 | 0 | NMIBC | High | Primary | Radical cystectomy | 2 | 1.795 |  |  | 71 | 0 |
| BC_T087 | F | 74 | a | 0 | 0 | NMIBC | High | Primary | Transurethral resection | 2 | 2.382 |  | C>T | 51 | 0 |
| BC_T088 | M | 81 | a | 0 | 0 | NMIBC | PUNLMP | Primary | Transurethral resection | 3 | 2.662 |  | C>T | 36 | 0 |
| BC_T089 | F | 75 | 1 | 0 | 0 | NMIBC | High | Primary | Transurethral resection | 2 | 1.747 |  | C>T | 34 | 0 |
| BC_T090 | M | 60 | 1 | 0 | 0 | NMIBC | High | Primary | Transurethral resection | 2 | 1.515 |  | C>T | 24 | 1 |
| BC_T091 | M | 75 | a | 0 | 0 | NMIBC | Low | Primary | Transurethral resection | 2 | 2.135 |  |  | 51 | 0 |
| BC_T092 | F | 81 | a | 0 | 0 | NMIBC | Low | Primary | Transurethral resection | 3 | 2.764 |  |  | 37 | 1 |
| BC_T093 | M | 55 | a | 0 | 0 | NMIBC | PUNLMP | Primary | Transurethral resection | 2 | 1.579 |  |  | 48 | 0 |
| BC_T094 | M | 77 | a | 0 | 0 | NMIBC | PUNLMP | Primary | Transurethral resection | 1 | 1.115 |  | C>T | 50 | 0 |
| BC_T095 | M | 62 | 2 | 0 | 0 | MIBC | High | Primary | Radical cystectomy | 2 | 2.038 |  |  | 74 | 0 |
| BC_T096 | M | 80 | 4 | 0 | 0 | MIBC | High | Primary | Radical cystectomy | 2 | 1.857 |  |  | 52 | 0 |
| BC_T097 | M | 73 | 1 | 0 | 0 | NMIBC | High | Primary | Transurethral resection | 3 | 3.019 | G>A |  | 35 | 1 |
| BC_T098 | M | 69 | a | 0 | 0 | NMIBC | Low | Primary | Transurethral resection | 2 | 2.025 | G>A |  | 63 | 0 |
| BC_T099 | M | 51 | 1 | 0 | 0 | NMIBC | High | Primary | Transurethral resection | 2 | 2.004 | G>A |  | 47 | 0 |
| BC_T100 | M | 74 | a | 0 | 0 | NMIBC | Low | Primary | Transurethral resection | 2 | 1.640 |  | C>T | 16 | 1 |
| BC_T101 | M | 67 | 1 | 0 | 0 | NMIBC | High | Primary | Transurethral resection | 2 | 1.787 | G>A | C>T | NA | NA |
| BC_T102 | M | 78 | 2 | 0 | 0 | MIBC | High | Primary | Radical cystectomy | 2 | 1.500 | G>A | C>T | 29 | 1 |
| BC_T103 | F | 40 | a | 0 | 0 | NMIBC | PUNLMP | Primary | Transurethral resection | 2 | 1.513 |  |  | 66 | 0 |
| BC_T104 | F | 65 | a | 0 | 0 | NMIBC | Low | Primary | Transurethral resection | 1 | 1.426 |  |  | 48 | 0 |
| BC_T105 | M | 64 | 1 | 0 | 0 | NMIBC | High | Primary | Radical cystectomy | 2 | 1.912 |  |  | 68 | 0 |
| BC_T106 | M | 47 | 1 | 0 | 0 | NMIBC | High | Primary | Transurethral resection | 2 | 1.766 |  |  | 54 | 0 |
| BC_T107 | M | 28 | a | 0 | 0 | NMIBC | PUNLMP | Primary | Transurethral resection | 2 | 1.508 |  |  | 67 | 0 |
| BC_T108 | M | 55 | a | 0 | 0 | NMIBC | Low | Primary | Transurethral resection | 2 | 2.418 |  |  | 48 | 0 |
| BC_T109 | M | 76 | 1 | 0 | 0 | NMIBC | High | Primary | Transurethral resection | 1 | 1.174 |  |  | 51 | 0 |
| BC_T110 | M | 57 | a | 0 | 0 | NMIBC | High | Primary | Transurethral resection | 2 | 2.494 |  | C>T | 37 | 1 |
| BC_T111 | M | 59 | a | 0 | 0 | NMIBC | PUNLMP | Primary | Transurethral resection | 2 | 1.698 |  |  | 52 | 1 |
| BC_T112 | F | 72 | a | 0 | 0 | NMIBC | Low | Primary | Transurethral resection | 2 | 1.709 |  | C>T | 47 | 0 |
| BC_T113 | M | 56 | a | 0 | 0 | NMIBC | PUNLMP | Primary | Transurethral resection | 1 | 1.245 |  |  | 62 | 0 |
| BC_T114 | M | 79 | 1 | 0 | 0 | NMIBC | Low | Primary | Transurethral resection | 2 | 1.610 |  |  | 41 | 1 |
| BC_T115 | M | 74 | a | 0 | 0 | NMIBC | Low | Primary | Transurethral resection | 1 | 0.968 |  |  | 54 | 0 |
| BC_T116 | M | 80 | 1 | 0 | 0 | NMIBC | High | Primary | Transurethral resection | 2 | 1.651 |  |  | 14 | 1 |
| BC_T117 | F | 73 | 1 | 0 | 0 | NMIBC | High | Primary | Transurethral resection | 1 | 1.107 | G>A |  | 37 | 1 |
| BC_T118 | M | 72 | a | 0 | 0 | NMIBC | PUNLMP | Primary | Transurethral resection | 1 | 1.301 | G>A |  | 52 | 0 |
| BC_T119 | M | 80 | 1 | 0 | 0 | NMIBC | High | Primary | Transurethral resection | 2 | 2.277 | G>A |  | 11 | 1 |
| BC_T120 | M | 80 | 1 | 0 | 0 | NMIBC | High | Primary | Transurethral resection | 1 | 1.332 | G>A |  | 18 | 1 |
| BC_T121 | M | 71 | 1 | 0 | 0 | NMIBC | High | Primary | Transurethral resection | 2 | 2.236 | G>A |  | 48 | 0 |
| BC_T122 | M | 61 | 1 | 0 | 0 | NMIBC | High | Primary | Transurethral resection | 1 | 1.328 | G>A |  | 43 | 1 |
| BC_T123 | F | 73 | 1 | 0 | 0 | NMIBC | High | Primary | Transurethral resection | 1 | 1.307 | G>A | C>T | 18 | 1 |
| BC_T124 | M | 73 | a | 0 | 0 | NMIBC | PUNLMP | Primary | Transurethral resection | 2 | 1.719 | G>A |  | 32 | 1 |
| BC_T125 | M | 55 | 4 | 1 | 0 | MIBC | High | Primary | Radical cystectomy | 2 | 1.686 |  |  | 67 | 0 |
| BC_T126 | F | 70 | a | 0 | 0 | NMIBC | Low | Primary | Transurethral resection | 1 | 1.319 | G>A |  | 17 | 0 |
| BC_T127 | M | 64 | a | 0 | 0 | NMIBC | Low | Primary | Transurethral resection | 2 | 1.653 | G>A |  | 27 | 0 |
| BC_T128 | M | 82 | 3 | 1 | 0 | MIBC | High | Primary | Radical cystectomy | 2 | 2.294 | G>A |  | 11 | 1 |
| BC_T129 | F | 63 | a | 0 | 0 | NMIBC | Low | Primary | Transurethral resection | 2 | 1.706 | G>A | C>T | 36 | 0 |
| BC_T130 | F | 54 | a | 0 | 0 | NMIBC | High | Primary | Transurethral resection | 3 | 2.553 | G>A |  | 43 | 0 |
| BC_T131 | F | 37 | 3 | 0 | 0 | MIBC | High | Primary | Radical cystectomy | 1 | 1.283 | G>A |  | NA | NA |
| BC_T132 | F | 72 | 2 | 0 | 0 | MIBC | High | Primary | Transurethral resection | 1 | 1.467 | G>A |  | 14 | 1 |
| BC_T133 | M | 56 | a | 0 | 0 | NMIBC | PUNLMP | Primary | Transurethral resection | 1 | 1.238 | G>A |  | 43 | 0 |
| BC_T134 | M | 49 | a | 0 | 0 | NMIBC | Low | Primary | Transurethral resection | 2 | 1.958 | G>A |  | 60 | 0 |
| BC_T135 | M | 34 | a | 0 | 0 | NMIBC | PUNLMP | Primary | Transurethral resection | 2 | 1.750 |  |  | 55 | 0 |
| BC_T136 | F | 57 | a | 0 | 0 | NMIBC | Low | Primary | Transurethral resection | 2 | 1.728 |  | C>T | 60 | 0 |
| BC_T137 | M | 75 | a | 0 | 0 | NMIBC | Low | Primary | Transurethral resection | 2 | 2.423 |  | C>T | 26 | 1 |
| BC_T138 | M | 79 | 1 | 0 | 0 | NMIBC | High | Primary | Transurethral resection | 2 | 1.784 |  | C>T | 77 | 0 |
| BC_T139 | M | 78 | 1 | 0 | 0 | NMIBC | High | Primary | Radical cystectomy | 2 | 2.244 |  |  | 38 | 1 |
| BC_T140 | M | 62 | a | 0 | 0 | NMIBC | High | Primary | Transurethral resection | 55 | 54.711 |  |  | 29 | 1 |
| BC_T141 | F | 64 | a | 0 | 0 | NMIBC | PUNLMP | Primary | Transurethral resection | 1 | 1.223 |  |  | 49 | 1 |
| BC_T142 | M | 73 | 2 | 0 | 0 | MIBC | High | Primary | Radical cystectomy | 2 | 1.505 |  |  | 48 | 1 |
| BC_T143 | F | 80 | 1 | 0 | 0 | NMIBC | High | Primary | Transurethral resection | 3 | 2.598 |  | C>T | 31 | 1 |
| BC_T144 | M | 74 | 2 | 0 | 0 | MIBC | High | Primary | Radical cystectomy | 2 | 1.779 |  |  | 37 | 1 |
| BC_T145 | F | 63 | 4 | 2 | 0 | MIBC | High | Primary | Radical cystectomy | 1 | 1.071 |  |  | 53 | 1 |
| BC_T146 | M | 37 | a | 0 | 0 | NMIBC | PUNLMP | Primary | Transurethral resection | 1 | 1.359 |  | C>T | 67 | 0 |
| BC_T147 | M | 67 | a | 0 | 0 | NMIBC | High | Primary | Transurethral resection | 1 | 1.416 |  |  | 52 | 0 |
| BC_T148 | M | 78 | a | 0 | 0 | NMIBC | High | Primary | Transurethral resection | 1 | 1.486 |  |  | 50 | 1 |
| BC_T149 | M | 61 | 1 | 0 | 0 | NMIBC | Low | Primary | Transurethral resection | 4 | 3.811 |  |  | 21 | 0 |
| BC_T150 | M | 83 | 1 | 0 | 0 | NMIBC | High | Primary | Radical cystectomy | 2 | 1.617 |  | C>T | 29 | 1 |
| BC_T151 | M | 80 | 1 | 0 | 0 | NMIBC | High | Primary | Transurethral resection | 4 | 4.090 |  | C>T | 29 | 1 |
| BC_T152 | F | 80 | 1 | 0 | 0 | NMIBC | High | Primary | Transurethral resection | 2 | 1.852 |  |  | 47 | 1 |
| BC_T153 | M | 56 | 4 | 2 | 0 | MIBC | High | Primary | Radical cystectomy | 1 | 1.396 |  |  | 15 | 1 |
| BC_T154 | M | 42 | 2 | 2 | 0 | MIBC | High | Primary | Radical cystectomy | 1 | 1.089 |  | C>T | 22 | 0 |
| BC_T155 | M | 70 | 3 | 1 | 0 | MIBC | High | Primary | Radical cystectomy | 2 | 1.669 | G>A | C>T | 18 | 0 |
| BC_T156 | F | 63 | 3 | 3 | 0 | MIBC | High | Primary | Radical cystectomy | 1 | 1.203 |  |  | 15 | 0 |
| BC_T157 | M | 27 | 1 | 0 | 0 | NMIBC | PUNLMP | Primary | Transurethral resection | 1 | 1.422 |  |  | 35 | 0 |
| BC_T158 | M | 59 | a | 0 | 0 | NMIBC | PUNLMP | Primary | Transurethral resection | 1 | 1.405 |  |  | 73 | 0 |
| BC_T159 | M | 75 | 1 | 0 | 0 | NMIBC | Low | Primary | Transurethral resection | 1 | 1.385 |  |  | 33 | 0 |
| BC_T160 | F | 79 | 2 | 0 | 0 | MIBC | High | Primary | Transurethral resection | 1 | 1.198 |  |  | 50 | 1 |
| BC_T161 | M | 67 | 1 | 0 | 0 | NMIBC | Low | Primary | Transurethral resection | 2 | 1.844 | G>A |  | 39 | 0 |
| BC_T162 | M | 71 | 1 | 0 | 0 | NMIBC | High | Primary | Radical cystectomy | 1 | 1.273 |  |  | 27 | 0 |
| BC_T163 | M | 66 | 4 | 0 | 0 | MIBC | High | Primary | Radical cystectomy | 1 | 1.385 |  |  | 49 | 1 |
| BC_T164 | M | 59 | 2 | 0 | 0 | MIBC | High | Primary | Transurethral resection | 2 | 1.515 |  |  | 37 | 1 |
| BC_T165 | M | 62 | 1 | 0 | 0 | NMIBC | Low | Primary | Transurethral resection | 1 | 1.293 |  |  | 58 | 0 |
| BC_T166 | M | 73 | 1 | 0 | 0 | NMIBC | Low | Primary | Transurethral resection | 2 | 1.860 |  |  | 63 | 0 |
| BC_T167 | M | 55 | 1 | 0 | 0 | NMIBC | Low | Primary | Transurethral resection | 2 | 1.962 |  |  | 61 | 0 |
| BC_T168 | M | 43 | 2 | 0 | 0 | MIBC | High | Primary | Radical cystectomy | 1 | 1.390 |  |  | 12 | 0 |
| BC_T169 | M | 83 | 3 | 0 | 0 | MIBC | High | Primary | Radical cystectomy | 2 | 1.681 |  |  | 11 | 1 |
| BC_T170 | M | 75 | 1 | 0 | 0 | NMIBC | High | Primary | Transurethral resection | 1 | 1.162 | G>A | C>T | 13 | 0 |
| BC_T171 | M | 68 | 3 | 2 | 0 | MIBC | High | Primary | Radical cystectomy | 2 | 1.593 |  |  | 26 | 1 |
| BC_T172 | F | 79 | a | 0 | 0 | NMIBC | PUNLMP | Primary | Transurethral resection | 2 | 2.487 | G>A |  | 29 | 0 |
| BC_T173 | M | 56 | 1 | 0 | 0 | NMIBC | Low | Primary | Transurethral resection | 1 | 1.059 | G>A | C>T | 33 | 0 |
| BC_T174 | M | 51 | 3 | 1 | 0 | MIBC | High | Primary | Radical cystectomy | 1 | 1.172 |  |  | 9 | 0 |
| BC_T175 | M | 34 | a | 0 | 0 | NMIBC | PUNLMP | Primary | Transurethral resection | 1 | 1.303 |  |  | 17 | 0 |
| BC_T176 | M | 53 | 3 | 0 | 0 | MIBC | High | Primary | Radical cystectomy | 9 | 9.136 |  |  | 20 | 1 |
| BC_T177 | M | 67 | 3 | 0 | 0 | MIBC | High | Primary | Radical cystectomy | 1 | 1.445 | G>A |  | 9 | 0 |
| BC_T178 | F | 56 | 3 | 0 | 0 | MIBC | High | Primary | Radical cystectomy | 1 | 0.939 |  | C>T | 10 | 0 |
| BC_T179 | M | 58 | 2 | 0 | 0 | MIBC | High | Primary | Radical cystectomy | 1 | 1.052 |  |  | 9 | 0 |
| BC_T180 | M | 31 | 1 | 0 | 0 | NMIBC | Low | Primary | Transurethral resection | 1 | 1.370 |  |  | 60 | 0 |
| BC_T181 | M | 52 | a | 0 | 0 | NMIBC | Low | Primary | Transurethral resection | 1 | 1.254 |  |  | 72 | 0 |
| BC_T182 | M | 64 | 2 | 0 | 0 | MIBC | High | Primary | Radical cystectomy | 1 | 1.153 |  |  | 31 | 0 |
| BC_T183 | M | 78 | 2 | 0 | 0 | MIBC | High | Primary | Radical cystectomy | 2 | 1.601 | G>A |  | 9 | 0 |
| BC_T184 | M | 66 | 3 | 2 | 0 | MIBC | High | Primary | Radical cystectomy | 2 | 1.919 |  |  | 11 | 1 |
| BC_T185 | M | 47 | 1 | 0 | 0 | NMIBC | Low | Primary | Transurethral resection | 1 | 1.111 | G>A | C>T | 23 | 0 |
| BC_T186 | M | 77 | 1 | 0 | 0 | NMIBC | High | Primary | Transurethral resection | 1 | 1.306 | G>A | C>T | 22 | 1 |
| BC_T187 | M | 74 | 1 | 0 | 0 | NMIBC | High | Primary | Radical cystectomy | 1 | 1.038 | G>A |  | 13 | 1 |
| BC_T188 | M | 76 | 3 | 1 | 0 | MIBC | High | Primary | Radical cystectomy | 1 | 1.379 | G>A |  | 9 | 1 |
| BC_T189 | M | 75 | 1 | 0 | 0 | NMIBC | Low | Primary | Transurethral resection | 2 | 1.867 | G>A | C>T | 12 | 0 |
| BC_T190 | M | 76 | 2 | 0 | 0 | MIBC | High | Primary | Radical cystectomy | 2 | 2.043 |  |  | NA | NA |
| BC_T191 | M | 55 | 3 | 1 | 0 | MIBC | High | Primary | Radical cystectomy | 1 | 1.300 |  |  | 8 | 1 |
| BC_T192 | M | 62 | 1 | 0 | 0 | NMIBC | Low | Primary | Transurethral resection | 1 | 1.370 | G>A |  | 17 | 0 |
| BC_T193 | M | 34 | 1 | 0 | 0 | NMIBC | PUNLMP | Primary | Transurethral resection | 1 | 1.209 | G>A |  | 16 | 0 |
| BC_T194 | M | 74 | 2 | 0 | 0 | MIBC | High | Primary | Radical cystectomy | 8 | 7.943 | G>A |  | 13 | 1 |
| BC_T195 | M | 82 | 2 | 0 | 0 | MIBC | High | Primary | Radical cystectomy | 3 | 2.654 | G>A |  | 11 | 1 |
| BC_T196 | M | 63 | 1 | 0 | 0 | NMIBC | Low | Primary | Transurethral resection | 1 | 1.404 | G>A | C>T | 32 | 0 |

^#^M, Male; F, Female.

^*^PUNLMP, papillary urothelial malignancy of low malignant potential; Low, low-grade papillary urothelial carcinoma; High, high-grade papillary urothelial carcinoma (WHO/ISUP 2004 classification).

^&^0 = survival; 1 = death; NA= not available.

**Supplementary Table 7. Correlation between *ADGRG6* enhancer mutation or *FRS2* copy number variation and clinicopathological features of UBC patients**

| Variable | *ADGRG6* enhancer mutation | | |  | *FRS2* copy number variation | | |
| --- | --- | --- | --- | --- | --- | --- | --- |
|  | No mutated | Mutated | *P* Value^#^ |  | Copy number ≤ 3 | Copy number > 3 | *P* Value^#^ |
| Age category |  |  | 0.024 |  |  |  | 0.938 |
| < 60 | 35 (37.2) | 23 (22.5) |  |  | 54 (29.7) | 4 (28.6) |  |
| ≥ 60 | 59 (62.8) | 79 (77.5) |  |  | 128 (70.3) | 10 (71.4) |  |
| Gender |  |  | 0.691 |  |  |  | > 0.999 |
| Male | 75 (79.8) | 79 (77.5) |  |  | 143 (78.6) | 11 (78.6) |  |
| Female | 19 (20.2) | 23 (22.5) |  |  | 39 (21.4) | 3 (21.4) |  |
| Histological grade |  |  | 0.887 |  |  |  | 0.935 |
| PUNLMP | 18 (19.1) | 17 (16.7) |  |  | 33 (18.1) | 2 (14.3) |  |
| Low | 24 (25.5) | 28 (27.5) |  |  | 48 (26.4) | 4 (28.6) |  |
| High | 52 (55.3) | 57 (55.9) |  |  | 101 (55.5) | 8 (57.1) |  |
| T status |  |  | 0.078 |  |  |  | 0.923 |
| Ta | 31 (33.0) | 41 (40.2) |  |  | 66 (36.3) | 6 (42.9) |  |
| T1 | 29 (30.9) | 39 (38.2) |  |  | 63 (34.6) | 5 (35.7) |  |
| T2 | 15 (16.0) | 14 (13.7) |  |  | 27 (14.8) | 2 (14.3) |  |
| T3 | 12 (12.8) | 7 (6.9) |  |  | 18 (9.9) | 1 (7.1) |  |
| T4 | 7 (7.4) | 1 (1.0) |  |  | 8 (4.4) | 0 (0) |  |
| N status |  |  | 0.224 |  |  |  | 0.241 |
| N- | 84 (89.4) | 96 (94.1) |  |  | 166 (91.2) | 14 (100) |  |
| N+ | 10 (10.6) | 6 (5.9) |  |  | 16 (8.8) | 0 (0) |  |
| Invasive stage |  |  | 0.024 |  |  |  | 0.539 |
| Non-muscle invasion | 60 (63.8) | 80 (78.4) |  |  | 129 (70.9) | 11 (78.6) |  |
| Muscle invasion | 34 (36.2) | 22 (21.6) |  |  | 53 (29.1) | 3 (21.4) |  |

All data are the number of patients (%).

^#^*P* values were calculated in GraphPad Prism (Version 7.00) using chi-square test. *P* values < 0.05 were considered to indicate statistical significance.

**Supplementary Table 8. Patient and tumor characteristics of the initial cohort of 65 UBCs and the additional cohort of 196 UBCs**

| Characteristic | The initial cohort (n=65) | The additional cohort (n=196) |
| --- | --- | --- |
| Age category | | |
| <60 | 21 (32.31) | 58 (29.59) |
| 60-69 | 27 (41.54) | 47 (23.98) |
| 70-79 | 13 (20.00) | 66 (33.67) |
| ≥ 80 | 4 (6.15) | 25 (12.76) |
| Gender | | |
| Male | 58 (89.23) | 154 (78.57) |
| Female | 7 (10.77) | 42 (21.43) |
| T status | | |
| Ta | 8 (12.31) | 72 (36.73) |
| T1 | 24 (36.92) | 68 (34.69) |
| T2 | 21 (32.31) | 29 (14.80) |
| T3 | 9 (13.85) | 19 (9.70) |
| T4 | 3 (4.61) | 8 (4.08) |
| *ADGRG6* enhancer mutation | | |
| G>A only | 11 (16.92) | 51 (26.02) |
| C>T/G only | 2 (3.08) | 32 (16.33) |
| Both | 2 (3.08) | 19 (9.69) |
| No mutation | 50 (76.92) | 94 (47.96) |
| *FRS2* copy number variation | | |
| Copy number ≤ 3 | 6 (9.23) | 14 (7.14) |
| Copy number > 3 | 59 (90.77) | 182 (92.86) |

All data are the number of patients (%).

**Supplementary Table 9**. Univariate and multivariate analysis of different prognostic parameters for UBC patients in the additional cohort

| Variable | Univariate Analysis | |  | | Multivariate Analysis | |
| --- | --- | --- | --- | --- | --- | --- |
|  | HR (95% CI)^#^ | *P* Value^*^ | | HR (95% CI)^#^ | | *P* Value^*^ |
| Gender (male versus female) | 1.3 (0.7-2.3) | 0.430 | | 1.6 (0.8-3.1) | | 0.147 |
| Age (< 60 years versus ≥ 60 years) | 2.8 (1.5-5.4) | 0.002 | | 2.5 (1.3-5.0) | | 0.008 |
| Histological grade (PUNLMP or Low versus High) | 5.2 (2.9-9.3) | <0.001 | | 3.6 (1.7-7.6) | | 0.001 |
| Invasive stage (non-muscle invasive versus muscle invasive) | 2.9 (1.8-4.7) | <0.001 | | 2.6 (1.1-6.4) | | 0.034 |
| T status (Ta versus T1 - T4) | 3.2 (1.8-5.4) | <0.001 | | 1.2 (0.6-2.5) | | 0.583 |
| N status (N0 versus N1 - N3) | 3.7 (1.8-7.5) | <0.001 | | 3.6 (1.5-8.6) | | 0.004 |
| Surgical approach (transurethral resection versus radical cystectomy) | 2.3 (1.4-3.7) | 0.001 | | 0.5 (0.2-1.4) | | 0.190 |
| *ADGRG6* enhancer mutation (no versus yes) | 2.2 (1.3-3.5) | 0.002 | | 3.0 (1.7-5.2) | | <0.001 |
| *FRS2* copy number variation (copy number ≤ 3 versus > 3 ) | 3.8 (2.0-7.4) | <0.001 | | 5.7 (2.8-11.5) | | <0.001 |

^#^ HRs (Hazard ratios) and 95% CIs (confidence intervals) were calculated using univariate or multivariate Cox proportional hazards regression in SPSS (Version 22).

^*^ *P* values were calculated using univariate or multivariate Cox proportional hazards regression in SPSS (Version 22). *P* values < 0.05 were considered to indicate statistical significance.

**Supplementary Table 10**. Kataegis events in the 65 UBC cases

| Sample | chr | kataegis_start | kataegis_end | SV_start | SV_end | gene |
| --- | --- | --- | --- | --- | --- | --- |
| BL01 | chr1 | 31,539,390 | 31,542,120 | 16,828,097 | 147,854,696 |  |
| BL02 | chr20 | 33,582,347 | 33,583,103 | 31,114,772 | 43,689,618 | *MYH7B* |
| BL02 | chr20 | 39,790,255 | 39,791,836 | 31,114,772 | 43,689,618 | *PLCG1* |
| BL04 | chr1 | 161,832,767 | 161,834,258 | 39,336,026 | 218,647,057 | *ATF6* |
| BL04 | chr1 | 56,006,508 | 56,008,854 | 39,336,026 | 218,647,057 | *DHCR24(Distal)* |
| BL04 | chr1 | 69,820,757 | 69,821,533 | 39,336,026 | 218,647,057 |  |
| BL06 | chr1 | 145,453,711 | 145,456,621 | 145,448,258 | 179,900,978 | *POLR3GL* |
| BL06 | chrX | 23,156,505 | 23,158,057 | 15,643,777 | 28,464,512 | *RP11-40F8.2* |
| BL07 | chr2 | 159,118,404 | 159,118,902 | 18,066,824 | 212,084,196 | *CCDC148* |
| BL07 | chr4 | 5,203,175 | 5,204,656 | 4,590,468 | 114,130,400 | *STK32B* |
| BL07 | chr4 | 5,205,885 | 5,207,761 | 5,206,221 | 5,379,081 | *STK32B* |
| BL07 | chr4 | 5,253,623 | 5,254,975 | 5,250,021 | 5,375,095 | *STK32B* |
| BL07 | chr4 | 5,257,812 | 5,259,066 | 5,250,021 | 5,375,095 | *STK32B* |
| BL07 | chr4 | 5,382,170 | 5,383,257 | 5,205,326 | 5,383,480 | *STK32B* |
| BL07 | chr4 | 113,142,077 | 113,145,868 | 4,590,468 | 114,130,400 |  |
| BL08 | chr19 | 10,042,603 | 10,044,839 | 5,412,838 | 10,917,917 | *OLFM2* |
| BL08 | chr19 | 21,123,609 | 21,125,873 | 21,124,295 | 21,131,712 | *ZNF85* |
| BL08 | chrX | 4,041,044 | 4,042,435 | 4,038,519 | 4,045,106 |  |
| BL09 | chr1 | 242,297,594 | 242,297,966 | 240,396,154 | 242,299,182 | *PLD5* |
| BL13 | chr1 | 4,243,651 | 4,249,590 | 4,248,020 | 4,409,034 |  |
| BL14 | chr11 | 68,815,368 | 68,816,893 | 57,430,485 | 93,963,585 | *TPCN2* |
| BL14 | chr11 | 71,946,678 | 71,948,835 | 57,430,485 | 93,963,585 | *INPPL1* |
| BL14 | chr12 | 112,327,492 | 112,330,353 | 5,209,173 | 122,993,756 | *MAPKAPK5* |
| BL14 | chr12 | 123,680,824 | 123,682,526 | 27,897,013 | 131,534,584 | *MPHOSPH9* |
| BL14 | chr17 | 55,435,632 | 55,437,389 | 55,435,893 | 55,442,394 | *MSI2* |
| BL14 | chr19 | 22,267,154 | 22,269,207 | 4,925,793 | 48,943,392 | *ZNF257* |
| BL14 | chr19 | 45,304,203 | 45,305,491 | 4,925,793 | 48,943,392 |  |
| BL14 | chr2 | 20,253,427 | 20,254,508 | 20,038,335 | 20,838,738 | *RP11-644K8.1* |
| BL14 | chr22 | 24,497,719 | 24,500,165 | 24,498,593 | 24,502,925 | *KB-318B8.7* |
| BL14 | chr22 | 24,501,422 | 24,503,311 | 24,498,593 | 24,502,925 | *CABIN1* |
| BL14 | chr5 | 6,434,587 | 6,436,198 | 6,426,311 | 6,435,948 |  |
| BL60 | chr1 | 32,358,679 | 32,360,867 | 32,172,387 | 46,833,987 | *RP11-84A19.4* |
| BL60 | chr11 | 31,794,988 | 31,796,798 | 30,441,681 | 32,486,501 | *ELP4* |
| BL60 | chr12 | 57,142,404 | 57,145,303 | 54,010,040 | 123,783,865 | *PRIM1* |
| BL60 | chr16 | 68,497,126 | 68,499,298 | 68,319,789 | 69,137,525 |  |
| BL60 | chr2 | 64,389,249 | 64,391,057 | 19,930,077 | 68,799,153 | *AC074289.1* |
| BL60 | chr3 | 161,374,466 | 161,377,106 | 161,368,209 | 161,376,015 |  |
| BL60 | chr5 | 131,974,380 | 131,976,300 | 131,425,329 | 132,117,538 | *RAD50* |
| BL60 | chr5 | 141,094,889 | 141,096,795 | 140,568,304 | 141,478,291 |  |
| BL16 | chr22 | 31,030,238 | 31,030,939 | 27,892,566 | 32,225,176 | *SLC35E4(Promoter)* |
| BL63 | chr3 | 174,395,970 | 174,396,849 | 174,381,052 | 174,397,589 | *NAALADL2* |
| BL63 | chr5 | 7,016,233 | 7,017,859 | 3,407,765 | 94,614,911 | *RP11-122F24.1* |
| BL21 | chr13 | 113,697,247 | 113,700,665 | 111,464,101 | 113,980,347 | *MCF2L* |
| BL21 | chr8 | 109,655,323 | 109,656,291 | 109,474,303 | 110,318,690 | *TMEM74* |
| BL49 | chr7 | 18,560,134 | 18,561,969 | 17,761,864 | 18,561,558 | *HDAC9* |
| BL24 | chr3 | 181,788,122 | 181,790,020 | 181,778,077 | 183,014,741 |  |
| BL51 | chr16 | 19,079,247 | 19,080,722 | 16,675,052 | 23,767,243 | *COQ7* |
| BL51 | chr6 | 105,181,512 | 105,182,426 | 105,179,119 | 105,183,119 | *HACE1* |
| BL52 | chr9 | 22,690,021 | 22,691,109 | 22,015,411 | 22,691,617 | *RP11-399D6.2* |
| BL25 | chr11 | 16,365,118 | 16,365,950 | 16,364,821 | 16,860,722 | *SOX6* |
| BL27 | chr10 | 26,312,955 | 26,313,847 | 26,047,589 | 52,076,716 | *MYO3A* |
| BL29 | chr1 | 159,838,472 | 159,840,272 | 159,327,883 | 161,685,206 | *RP11-190A12.7* |
| BL29 | chr11 | 28,186,757 | 28,188,065 | 28,019,521 | 28,356,203 | *METTL15* |
| BL29 | chr14 | 92,480,950 | 92,482,931 | 82,064,775 | 105,095,381 | *TRIP11* |
| BL29 | chr6 | 5,778,631 | 5,780,652 | 4,783,250 | 5,895,794 |  |
| BL56 | chr14 | 104,260,112 | 104,264,052 | 104,253,350 | 104,261,856 | *PPP1R13B* |
| BL57 | chrX | 16,990,193 | 16,991,289 | 16,987,030 | 17,001,693 | *REPS2* |
| BL35 | chr8 | 36,648,143 | 36,648,980 | 36,642,177 | 39,355,017 | *KCNU1* |
| BL36 | chr4 | 10,473,404 | 10,476,079 | 10,469,583 | 21,477,644 |  |
| BL58 | chr1 | 180,723,849 | 180,725,796 | 167,499,888 | 183,426,532 | *XPR1* |
| BL58 | chr10 | 22,209,416 | 22,210,761 | 22,208,991 | 22,212,991 | *DNAJC1* |
| BL58 | chr15 | 42,574,183 | 42,575,633 | 39,871,315 | 68,694,618 | *GANC* |
| BL58 | chr15 | 62,161,844 | 62,163,017 | 39,871,315 | 68,694,618 | *VPS13C* |
| BL58 | chr9 | 92,169,984 | 92,173,815 | 66,512,261 | 131,349,606 |  |
| BL37 | chr1 | 53,677,638 | 53,678,317 | 53,413,217 | 60,247,862 | *CPT2* |
| BL59 | chr2 | 150,334,729 | 150,336,581 | 149,306,619 | 232,281,584 |  |
| BL59 | chr2 | 171,530,295 | 171,532,593 | 149,306,619 | 232,281,584 | *AC007277.3* |
| BL41 | chr6 | 17,426,541 | 17,428,277 | 16,679,422 | 19,124,791 | *CAP2* |
| BL41 | chr8 | 124,324,384 | 124,326,034 | 118,470,145 | 126,873,574 |  |
| BL43 | chr2 | 74,679,048 | 74,681,089 | 66,331,181 | 75,588,167 |  |

Supplementary Table 11. Breakage-fusion-bridge events in the 65 UBC cases

| Sample | chr | inverion_start | inversion_end | cnv_start | cnv_end | cnv_num | telomere |
| --- | --- | --- | --- | --- | --- | --- | --- |
| BL02 | chr14 | 44,480,463 | 44,481,473 | 44,481,364 | 44,489,640 | 2.94963 | no |
| BL02 | chr14 | 49,977,817 | 49,983,412 | 49,976,007 | 49,982,257 | 3.14343 | no |
| BL02 | chr20 | 43,367,067 | 43,371,758 | 43,354,706 | 43,392,409 | 3.6294 | yes |
| BL02 | chr22 | 30,212,554 | 30,214,017 | 30,211,829 | 30,262,479 | 2.69663 | no |
| BL02 | chrX | 152,880,659 | 152,881,995 | 152,695,779 | 152,887,555 | 17.3849 | yes |
| BL02 | chrX | 153,047,746 | 153,051,739 | 153,046,736 | 153,059,720 | 17.681 | yes |
| BL03 | chr6 | 21,742,580 | 21,750,442 | 21,742,547 | 21,750,356 | 4.468 | no |
| BL04 | chr1 | 184,495,195 | 184,495,401 | 184,473,762 | 184,495,329 | 3.68247 | no |
| BL05 | chr12 | 67,659,237 | 67,662,032 | 67,542,896 | 67,662,982 | 2.0286 | no |
| BL05 | chr20 | 41,465,447 | 41,468,217 | 41,462,266 | 41,587,169 | 2.22614 | yes |
| BL05 | chr20 | 41,581,196 | 41,583,459 | 41,462,266 | 41,587,169 | 2.22614 | yes |
| BL05 | chr20 | 42,414,991 | 42,416,626 | 42,378,895 | 42,430,461 | 2.37305 | yes |
| BL05 | chr4 | 47,905,235 | 47,911,240 | 47,885,057 | 47,911,157 | 2.56411 | no |
| BL06 | chr1 | 160,371,429 | 160,373,444 | 160,370,982 | 160,373,421 | 2.86395 | no |
| BL06 | chr1 | 163,229,547 | 163,229,666 | 163,229,340 | 163,247,378 | 2.21097 | no |
| BL06 | chr2 | 64,223,128 | 64,224,578 | 64,215,091 | 64,240,164 | 2.25331 | no |
| BL06 | chr2 | 64,797,465 | 64,798,835 | 64,757,525 | 64,802,785 | 2.64454 | no |
| BL06 | chr2 | 79,047,496 | 79,065,259 | 79,041,471 | 79,113,558 | 2.66839 | no |
| BL06 | chr3 | 140,120,653 | 140,122,143 | 140,111,611 | 140,125,181 | 2.21973 | no |
| BL06 | chr3 | 154,512,934 | 154,513,350 | 154,504,753 | 154,516,431 | 3.03074 | no |
| BL06 | chr8 | 68,883,912 | 68,885,108 | 68,883,669 | 68,885,323 | 2.18812 | no |
| BL06 | chrX | 23,134,883 | 23,136,511 | 23,135,309 | 23,214,736 | 3.20113 | no |
| BL06 | chrX | 23,295,941 | 23,307,446 | 23,214,739 | 23,296,036 | 3.70133 | no |
| BL08 | chr1 | 150,195,236 | 150,206,806 | 150,206,698 | 150,221,826 | 2.91546 | no |
| BL08 | chr6 | 22,791,373 | 22,805,651 | 22,784,085 | 22,791,438 | 7.16589 | no |
| BL08 | chrX | 4,040,519 | 4,043,106 | 4,037,250 | 4,267,309 | 2.0982 | yes |
| BL09 | chr12 | 71,666,852 | 71,667,483 | 71,666,650 | 71,689,032 | 30.2811 | no |
| BL10 | chr12 | 24,852,869 | 24,856,114 | 24,814,840 | 24,853,666 | 5.9114 | no |
| BL10 | chr12 | 25,090,107 | 25,090,376 | 25,089,471 | 25,090,383 | 6.25132 | no |
| BL10 | chr8 | 122,122,295 | 122,124,163 | 122,122,333 | 122,212,921 | 2.39935 | yes |
| BL10 | chr8 | 122,202,225 | 122,202,831 | 122,122,333 | 122,212,921 | 2.39935 | yes |
| BL10 | chr8 | 127,745,964 | 127,748,299 | 127,727,832 | 127,749,727 | 2.06549 | yes |
| BL10 | chr8 | 135,866,044 | 135,867,426 | 135,867,259 | 135,885,900 | 3.03143 | yes |
| BL10 | chr8 | 135,935,271 | 135,935,824 | 135,927,836 | 135,948,114 | 2.75691 | yes |
| BL10 | chr8 | 136,617,988 | 136,618,640 | 136,613,315 | 136,650,658 | 3.75589 | yes |
| BL10 | chr8 | 137,082,932 | 137,084,126 | 137,082,635 | 137,084,159 | 3.41086 | yes |
| BL10 | chr8 | 138,078,721 | 138,086,004 | 138,077,353 | 138,090,892 | 3.58044 | yes |
| BL10 | chr8 | 138,695,401 | 138,695,890 | 138,695,393 | 138,708,910 | 5.61389 | yes |
| BL10 | chr8 | 138,726,094 | 138,727,891 | 138,723,655 | 138,766,466 | 4.83575 | yes |
| BL21 | chr13 | 113,906,350 | 113,916,369 | 113,905,455 | 113,906,599 | 4.09855 | yes |
| BL21 | chr13 | 115,079,434 | 115,094,281 | 115,027,345 | 115,080,395 | 8.87749 | yes |
| BL22 | chr8 | 86,995,717 | 86,996,103 | 86,990,545 | 87,012,252 | 3.12231 | no |
| BL29 | chr1 | 54,705,354 | 54,707,778 | 54,629,299 | 54,708,103 | 2.33839 | no |
| BL29 | chr1 | 60,418,074 | 60,419,419 | 60,417,845 | 60,421,280 | 2.46606 | no |
| BL29 | chr2 | 56,217,236 | 56,219,598 | 56,114,308 | 56,217,815 | 2.1611 | no |
| BL29 | chr2 | 64,890,761 | 64,891,530 | 64,890,477 | 64,941,568 | 3.24354 | no |
| BL29 | chr2 | 65,501,847 | 65,505,098 | 65,496,040 | 65,504,946 | 2.49126 | no |
| BL29 | chr3 | 9,489,253 | 9,491,240 | 9,490,930 | 9,602,869 | 2.86501 | yes |
| BL29 | chr6 | 21,155,617 | 21,162,663 | 21,153,992 | 21,172,907 | 3.08457 | no |
| BL31 | chr10 | 8,291,543 | 8,310,406 | 8,285,992 | 8,292,785 | 2.65227 | yes |
| BL31 | chr12 | 69,159,024 | 69,162,938 | 69,143,368 | 69,159,764 | 12.4791 | no |
| BL32 | chr12 | 117,016,416 | 117,017,286 | 116,994,954 | 117,052,510 | 10.3924 | yes |
| BL32 | chr16 | 24,373,853 | 24,375,169 | 24,373,688 | 24,375,239 | 2.75975 | no |
| BL32 | chr5 | 59,588,392 | 59,601,177 | 59,587,087 | 59,588,557 | 2.24849 | no |
| BL39 | chr11 | 69,905,863 | 69,907,989 | 69,804,001 | 69,933,054 | 15.8226 | no |
| BL40 | chr6 | 23,345,343 | 23,349,051 | 23,344,525 | 23,348,990 | 2.25715 | no |
| BL42 | chr19 | 39,310,467 | 39,312,123 | 39,232,976 | 39,311,926 | 2.39605 | no |
| BL43 | chr1 | 170,823,894 | 170,825,354 | 170,810,614 | 170,827,252 | 2.47159 | no |
| BL43 | chr10 | 1,227,604 | 1,230,933 | 1,230,537 | 1,238,883 | 2.12815 | yes |
| BL43 | chr7 | 17,634,943 | 17,649,829 | 17,633,554 | 17,635,169 | 3.36395 | no |
| BL43 | chr7 | 19,794,048 | 19,797,880 | 19,742,016 | 19,797,991 | 4.0835 | no |
| BL47 | chr3 | 3,007,433 | 3,009,133 | 2,890,688 | 3,009,066 | 2.35625 | yes |
| BL47 | chr3 | 8,922,539 | 8,925,722 | 8,918,279 | 8,965,108 | 3.4808 | yes |
| BL47 | chr3 | 10,639,586 | 10,641,556 | 10,566,113 | 10,640,968 | 4.18052 | yes |
| BL47 | chr3 | 13,507,365 | 13,508,869 | 13,333,837 | 13,507,479 | 4.70727 | yes |
| BL47 | chr3 | 17,475,976 | 17,477,916 | 17,475,793 | 17,477,836 | 2.58233 | yes |
| BL47 | chr3 | 75,271,639 | 75,273,595 | 75,273,536 | 75,274,194 | 2.04782 | no |
| BL49 | chr11 | 80,527,517 | 80,529,516 | 80,504,367 | 80,529,692 | 4.51549 | no |
| BL49 | chr11 | 81,131,051 | 81,132,569 | 81,130,291 | 81,132,477 | 6.16909 | no |
| BL49 | chr11 | 84,620,742 | 84,624,530 | 84,620,660 | 84,624,476 | 2.50326 | no |
| BL58 | chr1 | 160,007,983 | 160,022,032 | 160,021,937 | 160,073,270 | 3.11398 | no |
| BL58 | chr12 | 52,290,865 | 52,304,084 | 52,288,508 | 52,394,842 | 2.693 | no |
| BL63 | chr1 | 22,634,729 | 22,635,590 | 22,634,510 | 22,705,628 | 3.59652 | no |
| BL63 | chr1 | 158,801,674 | 158,811,195 | 158,797,161 | 158,801,937 | 6.16299 | no |
| BL63 | chr1 | 161,089,364 | 161,089,538 | 161,083,902 | 161,094,457 | 2.94473 | no |
| BL63 | chr17 | 36,071,941 | 36,080,701 | 36,071,459 | 36,110,521 | 2.92385 | no |
| BL63 | chr17 | 36,932,939 | 36,934,897 | 36,932,723 | 36,963,080 | 7.60148 | no |
| BL63 | chr17 | 37,610,014 | 37,611,685 | 37,609,002 | 37,645,547 | 8.86019 | no |
| BL63 | chr5 | 3,179,074 | 3,180,497 | 3,174,425 | 3,236,334 | 3.40924 | yes |
| BL63 | chr6 | 19,861,765 | 19,863,249 | 19,862,209 | 19,863,274 | 3.93332 | no |
| BL63 | chr6 | 19,957,429 | 19,968,095 | 19,957,332 | 19,968,594 | 4.70636 | no |
| BL63 | chr6 | 20,494,968 | 20,498,680 | 20,464,447 | 20,495,448 | 3.55622 | no |
| BL63 | chr6 | 22,388,869 | 22,390,039 | 22,349,693 | 22,389,480 | 2.91734 | no |

**Supplementary Table 12. Primers and siRNAs used in this study**

| siRNA/Genes | Sense/Primer-F（5'-3'） | Antisense/Primer-R（5'-3'） | Usage |
| --- | --- | --- | --- |
| siADGRG6 #1 | GUUCUUAUUUGCUUUAUAUAU | AUAUAAAGCAAAUAAGAACAG | Knockdown of target genes |
| siADGRG6 #2 | GGAAUUAUCUAUAGAAUAUCC | AUAUUCUAUAGAUAAUUCCAU |
| siFRS2 | CUAAAUGGCUACCAUAAUAAU | UAUUAUGGUAGCCAUUUAGAG |
| siCon | UUCUCCGAACGUGUCACGUTT | ACGUGACACGUUCGGAGAATT |
| *ZFP36L1* | TTCCACACCATCGGCTTTTG | TAAAGCTATGCTGGAGGCGG | Quantitative RT-PCR |
| *ELF3* | GCAACATGACCTACGAGAAGC | CGACTCTGGAGAACCTCTTCC |
| *ADGRG6* | TCACACCTTCTGAGTACCGC | AGTTCTGCCCATCAGCAGTT |
| *FRS2* | AAGCCCGCAAGCTAAGTAGG | GCACTTGCTGGCACTGTTAC |
| *GAPDH* | GAAATCCCATCACCATCTTCCAGG | GAGCCCCAGCCTTCTCCATG |
| *ADGRG6* | TTCCAGCAACCCCCAAGAAA | CAGCTCCACTCCACCAAGTC | Sanger sequencing and q-PCR |
| *FRS2* | ATGGGAATGAGTTAGGTTCTGGC | GCGGGTGTATAAAATCAGTTCTGTG |
| *GAPDH* | CATGTTCCAATATGATTCCAC | CCTGGAAGATGGTGATG |
