# Supplementary material for: Whole-genome sequencing identifies ADGRG6 enhancer mutations and FRS2 duplications as angiogenesis-related drivers in bladder cancer
Source: Nat Commun. 2019 Feb 12;10:720. doi: 10.1038/s41467-019-08576-5 (PMC6372626; doi:10.1038/s41467-019-08576-5)
Supplement: Supplementary file 2 — Description of Additional Supplementary Files [file 41467_2019_8576_MOESM2_ESM.pdf]

Description of Additional Supplementary Files

File Name: Supplementary Data 1
Description: Summary of all kinds of somatic mutations in the 65 UBC cases

File Name: Supplementary Data 2
Description: Structural variations in the 65 UBC cases

File Name: Supplementary Data 3
Description: Fusion events in the 65 UBC cases

File Name: Supplementary Movie 1
Description: A movie displays the high level of microvessel density in bladder tumor
